# Impact of the COVID-19 pandemic on surgical care in the Netherlands

**DOI:** 10.1093/bjs/znac301

**Published:** 2022-09-06

**Authors:** Michelle R de Graaff, Rianne N M Hogenbirk, Yester F Janssen, Arthur K E Elfrink, Ronald S L Liem, Simon W Nienhuijs, Jean-Paul P M de Vries, Jan-Willem Elshof, Emiel Verdaasdonk, Jarno Melenhorst, H L van Westreenen, Marc G H Besselink, Jelle P Ruurda, Mark I van Berge Henegouwen, Joost M Klaase, Marcel den Dulk, Mark van Heijl, Johannes H Hegeman, Jerry Braun, Daan M Voeten, Franka S Würdemann, Anne-Loes K Warps, Anna J Alberga, J Annelie Suurmeijer, Erman O Akpinar, Nienke Wolfhagen, Anne Loes van den Boom, Marieke J Bolster-van Eenennaam, Peter van Duijvendijk, David J Heineman, Michel W J M Wouters, Schelto Kruijff, Dutch CovidSurg Collaborative Study Group, J N Helleman, C L Koningswoud-Terhoeve, E Belt, J A B van der Hoeven, G M H Marres, F Tozzi, E M von Meyenfeldt, R R J Coebergh-van den Braak, S Huisman, A M Rijken, R Balm, F Daams, C Dickhoff, W J Eshuis, S S Gisbertz, H R Zandbergen, K J Hartemink, S A Keessen, N F M Kok, K F D Kuhlmann, J W van Sandick, A A Veenhof, A Wals, M S van Diepen, L Schoonderwoerd, C T Stevens, D Susa, B L W Bendermacher, N Olofsen, M van Himbeeck, I H J T de Hingh, H J B Janssen, M D P Luyer, G A P Nieuwenhuijzen, M Ramaekers, R Stacie, A K Talsma, M W Tissink, D Dolmans, R Berendsen, J Heisterkamp, W A Jansen, M de Kort-van Oudheusden, R M Matthijsen, D J Grünhagen, S M Lagarde, A P W M Maat, P C van der Sluis, R B Waalboer, V Brehm, J P van Brussel, M Morak, E D Ponfoort, J E M Sybrandy, P L Klemm, W Lastdrager, H W Palamba, S M van Aalten, L N L Tseng, K E A van der Bogt, W J de Jong, J W A Oosterhuis, Q Tummers, G M van der Wilden, S Ooms, E H Pasveer, H T C Veger, M J Molegraafb, V B Nieuwenhuijs, G A Patijn, M E V van der Veldt, D Boersma, S T W van Haelst, I D van Koeverden, M L Rots, B A Bonsing, N Michiels, O D Bijlstra, J Braun, D Broekhuis, H W Brummelaar, H H Hartgrink, A Metselaar, J S D Mieog, I B Schipper, W O de Steur, B Fioole, E C Terlouw, C Biesmans, J W A M Bosmans, S A W Bouwense, S H E M Clermonts, M M E Coolsen, B M E Mees, G W H Schurink, J W Duijff, T van Gent, L C F de Nes, D Toonen, M J Beverwijk, E van den Hoed, B Keizers, W Kelder, B P J A Keller, B B Pultrum, E van Rosum, A G Wijma, F van den Broek, W K G Leclercq, M J A Loos, J M L Sijmons, R H D Vaes, P J Vancoillie, E C J Consten, J M J Jongen, P M Verheijen, V van Weel, C H P Arts, J Jonker, G Murrmann-Boonstra, J P E N Pierie, J Swart, E B van Duyn, R H Geelkerken, R de Groot, N L Moekotte, A Stam, A Voshaar, G J D van Acker, R M A Bulder, D J Swank, I T A Pereboom, W H Hoffmann, M Orsini, J J Blok, J H P Lardenoije, M M P J Reijne, P van Schaik, L Smeets, S M M van Sterkenburg, N J Harlaar, S Mekke, T Verhaakt, E Cancrinus, G W van Lammeren, I Q Molenaar, H C van Santvoort, A W F Vos, A P Schouten- van der Velden, K Woensdregt, S P Mooy-Vermaat, D M Scharn, H A Marsman, F Rassam, F R Halfwerk, A J Andela, C I Buis, G M van Dam, K ten Duis, B van Etten, L Lases, M Meerdink, V E de Meijer, B Pranger, S Ruiter, M Rurenga, A Wiersma, A R Wijsmuller, K I Albers, P B van den Boezem, B Klarenbeek, B M van der Kolk, C J H M van Laarhoven, E Matthée, N Peters, C Rosman, A M A Schroen, M W J Stommel, A F T M Verhagen, R van der Vijver, M C Warlé, J H W de Wilt, J W van den Berg, T Bloemert, G J de Borst, E H van Hattum, C E V B Hazenberg, J A van Herwaarden, R van Hillegerberg, T E Kroese, B J Petri, R J Toorop, F Aarts, R J L Janssen, S H P Janssen-Maessen, M Kool, H Verberght, D E Moes, J W Smit, A M Wiersema, B P Vierhout, B de Vos, F C den Boer, N A M Dekker, J M J Botman, M J van Det, E C Folbert, E de Jong, J C Koenen, E A Kouwenhoven, I Masselink, L H Navis, H J Belgers, M N Sosef, J H M B Stoot

**Affiliations:** Department of Surgery, University Medical Centre Groningen, Groningen, the Netherlands; Dutch Institute for Clinical Auditing, Scientific Bureau, Leiden, the Netherlands; Department of Surgery, Maastricht University Medical Centre, Maastricht, the Netherlands; Department of Surgery, Gelre Ziekenhuizen, Apeldoorn, the Netherlands; Department of Surgery, University Medical Centre Groningen, Groningen, the Netherlands; Department of Surgery, University Medical Centre Groningen, Groningen, the Netherlands; Department of Surgery, Amsterdam UMC location Vrije Universiteit Amsterdam, Amsterdam, the Netherlands; Department of Surgery, Dutch Obesity Clinic, Gouda, the Netherlands; Department of Surgery, Groene Hart Hospital, Gouda, the Netherlands; Department of Surgery, Catharina Hospital, Eindhoven, the Netherlands; Department of Surgery, University Medical Centre Groningen, Groningen, the Netherlands; Department of Surgery, VieCuri Medical Centre, Venlo, the Netherlands; Department of Surgery, Jeroen Bosch Hospital, ‘s Hertogenbosch, the Netherlands; Department of Surgery, Maastricht University Medical Centre, Maastricht, the Netherlands; Department of Surgery, Isala, Zwolle, the Netherlands; Department of Surgery, Amsterdam UMC, Location University of Amsterdam, Amsterdam, the Netherlands; Cancer Centre Amsterdam, Amsterdam, the Netherlands; Department of Surgery, University Medical Centre Utrecht, Utrecht, the Netherlands; Department of Surgery, Amsterdam UMC, Location University of Amsterdam, Amsterdam, the Netherlands; Department of Surgery, University Medical Centre Groningen, Groningen, the Netherlands; Department of Surgery, Maastricht University Medical Centre, Maastricht, the Netherlands; Department of Surgery, Diakonessenhuis, Utrecht, the Netherlands; Department of Surgery, Ziekenhuisgroep Twente Almelo-Hengelo, Almelo, Hengelo, the Netherlands; Department of Surgery, Leiden University Medical Centre, Leiden, the Netherlands; Dutch Institute for Clinical Auditing, Scientific Bureau, Leiden, the Netherlands; Department of Surgery, Amsterdam UMC location Vrije Universiteit Amsterdam, Amsterdam, the Netherlands; Dutch Institute for Clinical Auditing, Scientific Bureau, Leiden, the Netherlands; Department of Surgery, Leiden University Medical Centre, Leiden, the Netherlands; Dutch Institute for Clinical Auditing, Scientific Bureau, Leiden, the Netherlands; Department of Surgery, Leiden University Medical Centre, Leiden, the Netherlands; Dutch Institute for Clinical Auditing, Scientific Bureau, Leiden, the Netherlands; Department of Vascular Surgery, Erasmus MC, Rotterdam, the Netherlands; Dutch Institute for Clinical Auditing, Scientific Bureau, Leiden, the Netherlands; Department of Surgery, Amsterdam UMC, Location University of Amsterdam, Amsterdam, the Netherlands; Cancer Centre Amsterdam, Amsterdam, the Netherlands; Dutch Institute for Clinical Auditing, Scientific Bureau, Leiden, the Netherlands; Department of Surgery, Maastricht University Medical Centre, Maastricht, the Netherlands; Dutch Institute for Clinical Auditing, Scientific Bureau, Leiden, the Netherlands; Department of Surgery, Radboud University Medical Centre, Nijmegen, the Netherlands; Department of Surgery, University Medical Centre Groningen, Groningen, the Netherlands; Department of Surgery, Gelre Ziekenhuizen, Apeldoorn, the Netherlands; Department of Surgery, Gelre Ziekenhuizen, Apeldoorn, the Netherlands; Department of Surgery, Amsterdam UMC location Vrije Universiteit Amsterdam, Amsterdam, the Netherlands; Dutch Institute for Clinical Auditing, Scientific Bureau, Leiden, the Netherlands; Department of Biomedical Data Sciences, Leiden University Medical Centre, Leiden, the Netherlands; Department of Surgical Oncology, Netherlands Cancer Institute-Antoni van Leeuwenhoek, Amsterdam, the Netherlands; Department of Surgery, University Medical Centre Groningen, Groningen, the Netherlands

## Abstract

**Background:**

The COVID-19 pandemic caused disruption of regular healthcare leading to reduced hospital attendances, repurposing of surgical facilities, and cancellation of cancer screening programmes. This study aimed to determine the impact of COVID-19 on surgical care in the Netherlands.

**Methods:**

A nationwide study was conducted in collaboration with the Dutch Institute for Clinical Auditing. Eight surgical audits were expanded with items regarding alterations in scheduling and treatment plans. Data on procedures performed in 2020 were compared with those from a historical cohort (2018–2019). Endpoints included total numbers of procedures performed and altered treatment plans. Secondary endpoints included complication, readmission, and mortality rates.

**Results:**

Some 12 154 procedures were performed in participating hospitals in 2020, representing a decrease of 13.6 per cent compared with 2018–2019. The largest reduction (29.2 per cent) was for non-cancer procedures during the first COVID-19 wave. Surgical treatment was postponed for 9.6 per cent of patients. Alterations in surgical treatment plans were observed in 1.7 per cent. Time from diagnosis to surgery decreased (to 28 days in 2020, from 34 days in 2019 and 36 days in 2018; *P* < 0.001). For cancer-related procedures, duration of hospital stay decreased (5 *versus* 6 days; *P* < 0.001). Audit-specific complications, readmission, and mortality rates were unchanged, but ICU admissions decreased (16.5 *versus* 16.8 per cent; *P* < 0.001).

**Conclusion:**

The reduction in the number of surgical operations was greatest for those without cancer. Where surgery was undertaken, it appeared to be delivered safely, with similar complication and mortality rates, fewer admissions to ICU, and a shorter hospital stay.

## Teaser

During the COVID-19 pandemic, a 13.6 per cent reduction in the number of surgical procedures performed was observed in 2020. Despite great pressure on healthcare, the COVID-19 pandemic did not cause an increase in adverse surgical outcomes, and oncological surgery-related duration of hospital and ICU stay were significantly shorter.

## Introduction

Since the onset of the COVID-19 pandemic in December 2019, 505 million infections and 6.2 million deaths have been reported worldwide^1^. Moreover, an estimated 180 000 healthcare workers have been reported to have died from COVID-19 between January 2020 and May 2021^2^. With these large numbers, COVID-19 has caused major disruption to regular healthcare, with serious consequences for patients both with and without COVID-19.

To meet the increased demand for COVID-19-related healthcare and ICU capacity, hospitals were forced to reduce and adjust the level of regular healthcare. For example, most health institutions adopted a crisis strategy that involved the reallocation of personnel, repurposing surgical theatres and postoperative recovery areas as ICUs, and reducing hospital attendances by postponing outpatient clinic appointments. Furthermore, temporary cancellation of cancer screening programmes was observed on a national scale^3,4^.

Although the COVID-19 pandemic affected multiple facets of healthcare, elective planned surgery is one of the fields that witnessed the greatest curtailment by many institutions, resulting in a weekly decrease of 2.4 million elective surgical procedures globally^5^. However, the impact of the deprioritization and cancellation of elective surgical care is still relatively unknown. It has been hypothesized that, because of this strategy, surgical waiting lists have expanded, leading to more patients visiting emergency departments or presenting with an advanced stage of disease, compared with the pre-COVID-19 era. Hypothetically, this may have resulted in dismal surgical outcomes and an inferior prognosis^5^.

To date, limited data have been available regarding the broad consequences of COVID-19 in terms of general surgical care on a national basis^6^. Therefore, data from several Dutch surgical audits, covering different surgical specialties, were analysed to gain insights into the true impact of the pandemic on elective surgical procedures in the Netherlands in 2020. The aim of this study was to determine the impact of COVID-19 on surgical care in the Netherlands, expressed as the number of procedures performed, altered treatment plans, and surgical outcomes, during 2020.

## Methods

### Study design

This nationwide prospective cohort study was performed in collaboration with the Dutch Institute for Clinical Auditing (DICA), an organization that facilitates clinical auditing using a validated process of systematic analysis of the quality of care^7,8^.

From August 2020, surgical audits for lung cancer surgery (Dutch Lung Cancer Audit - Surgery - DLCA-S), upper gastrointestinal cancer surgery (Dutch Upper-GI Cancer Audit - DUCA), pancreatic cancer surgery (Dutch Pancreatic Cancer Audit - DPCA), hepatobiliary surgery (Dutch Hepato Biliary Audit - DHBA), colorectal cancer surgery (Dutch Colorectal Cancer Audit - DCRA), hip fracture surgery (Dutch Hip Fracture Audit - DHFA), aortic aneurysm surgery (Dutch Surgical Aneurysm Audit - DSAA), and bariatric surgery (Dutch Audit for Treatment of Obesity - DATO) were expanded with an additional COVID-19 survey. This survey focused on alterations or delays in treatment and diagnosis, and perioperative outcomes during the pandemic.

All hospitals in the Netherlands performing pulmonary, upper gastrointestinal, pancreatic, hepatobiliary, colorectal, hip fracture, aneurysmal or bariatric procedures were approached to participate in the Dutch COVIDSurg II Snapshot Study. Participating hospitals provided written consent to participate. Under Dutch law, no ethical approval was required as only fully anonymized data were available for the purpose of this study.

### Patient selection

All patients who underwent pulmonary, upper gastrointestinal, pancreatic, hepatobiliary, colorectal, hip fracture, aneurysm or bariatric surgery in one of the participating hospitals were included. Patients who underwent surgery between 1 January 2018 and 31 December 2019 were included in the historical cohort, and those who underwent a surgical procedure between 1 January 2020 and 31 December 2020 were included in the study group.

### Data collection

Local investigators in each participating hospital were responsible for data collection from electronic patient records in prospectively maintained web-based audit databases (*Appendix S1*).

### Study endpoints

It was hypothesized that the total number of surgical procedures decreased in 2020, and that the possible expansion of surgical waiting lists might have led to increased emergency department visits, diseases at more advanced stages, and so dismal surgical outcomes. Therefore, the primary endpoints included the total number of surgical procedures performed in comparison with the mean for the historical cohort treated in 2018–2019, as well as the number of altered treatment plans noted during the COVID-19 pandemic. Secondary endpoints were changes in postoperative outcomes, including duration of hospital stay (interval between day of hospital admission or day of index surgery and day of discharge from hospital), readmissions to hospital within 30 days of discharge, severe complications (defined as need for reintervention or ICU admission and/or causing death (Clavien–Dindo grade IIIa or higher))^9^, ICU admission (yes/no), duration of ICU stay, and mortality rate, which was based on postoperative deaths within 30 or 90 days after surgery or in-hospital mortality, depending on availability of data in specific audits (*Table S1* and *Appendix S2*). Numbers of acute and elective surgical procedures were compared with those in the historical cohort. Acute surgical procedures were defined as procedures planned within 72 h of first surgical presentation.

Altered treatment plans were defined by a difference in time to surgery (calculated as number of days between the first (physical or video–telephone) appointment at the outpatient clinic and the surgical procedure (except for hip fracture surgery, for which time to surgery was calculated as the interval between day of presentation at the emergency department and day of surgery)), delay to surgery perceived by the surgeon (either because of a COVID-19 infection, reduction in hospital capacity, or for other reasons), or COVID-19-related changes in surgical approach or neoadjuvant treatment plan. Data on changes in treatment plans were collected through the additional COVID-19 survey.

### Variables

Variables registered in all the included audits were demographics, such as age and sex, and presence of co-morbidities. An overview of variables that were available in only a selection of audits are outlined in *Table S1* and *Appendix S2*.

### COVID-19 waves

A COVID-19 wave was defined as a time interval with a high incidence rate of SARS-CoV-2 viral infections in the Netherlands. According to the National Institute for Public Health and the Environment, the first COVID-19 wave in this study occurred between 16 March and 24 May 2020, and the second wave between 21 September 2020 and 14 July 2021. However, because this study included only patients who underwent surgical procedures in 2020, the second COVID-19 wave was considered to have ended on 31 December 2020 in this study^10^. The interval between the two COVID-19 waves is referred to as the interim period.

### Statistical analysis

Descriptive statistics were used to compare demographics, surgical details, and surgical outcomes of 2020 with mean values for 2018 and 2019. The number of procedures carried out and the surgical outcomes of 2020 were analysed separately for both COVID-19 waves and the interim period, and were compared with a historical cohort in equivalent time intervals (2018–2019). Analyses were performed on data from all surgical audits combined and on audit-specific data.

Categorical variables are presented as numbers with percentages, and were compared using Pearson’s χ^2^ test. Normally distributed variables are presented as mean (s.d.), and those with a skewed distribution as median (i.q.r.); they were analysed using Student’s *t* test or the Mann–Whitney *U* test depending on the distribution. Missing data up to 15 per cent were excluded from the statistical analyses. When missing data exceeded 15 per cent of the total group, the missing data were analysed as a separate group. Non-registered data were excluded from the analyses. Both missing and non-registered data are presented separately in the tables. Given the major differences between the different clinical audits, subanalyses were undertaken to assess the differences between oncological and non-oncological procedures as well as acute and elective procedures. All statistical analyses were done using R version 4.0.0 (R Project for Statistical Computing, Vienna, Austria).

## Results

Some 40 296 patients from eight surgical audits, covering the interval from 2018 to 2020, in 50 Dutch hospitals, were included in this study (*Fig. S1*, *Appendices S3* and *S4*). Of these 40 296 patients, 13 985 (34.7 per cent) were treated surgically in 2018, 14 157 (35.1 per cent) in 2019, and 12 154 (30.2 per cent) in 2020. Of patients treated in 2020, 2133 (17.5 per cent) had surgical treatment for lung cancer, 931 (7.7 per cent) for upper gastrointestinal cancer, 663 (5.5 per cent) for pancreatic cancer, 1215 (10.0 per cent) for hepatobiliary diseases, 1933 (15.9 per cent) for colorectal diseases, 2138 (17.6 per cent) for hip fractures, 1595 (13.1 per cent) for abdominal aneurysms, and 1543 (12.7 per cent) underwent a bariatric procedure. Of all the patients included in 2020, 5494 (45.2 per cent) underwent surgery because of a malignancy.

### Demographics

In 2020, the median age was 69 (i.q.r. 57–77) years, which was higher than that reported for the 2018 and 2019 cohorts (67 (55–76) years for both; *P* < 0.001), and 53.5 per cent of the patients were men (*Table 1*). Compared with the historical cohort, a smaller proportion of patients had a BMI above 30 kg/m^2^ (20.3 per cent in 2020 *versus* 25.3 per cent in 2019 and 26.3 per cent in 2018; *P* < 0.001). Of those operated on in 2020, co-morbidities were present in 45.2 per cent (44.4 per cent in 2019 and 28.3 per cent in 2018; *P* < 0.001) and 42.3 per cent had an ASA grade of III or higher (43.3 per cent in 2019 and 39.8 per cent in 2018; *P* < 0.001). Moreover, in 2020, 75 surgically treated patients (0.6 per cent) had a confirmed COVID-19 infection. Audit-specific demographics can be found in *Tables S2a–h* and *Appendix S2*.

**Table 1:** Overview of characteristics of all included patients undergoing surgery between 1 January 2018 and 31 December 2020

	2018 (*n* = 13 985)	2019 (*n* = 14 157)	2020 (*n* = 12 154)	*P*†
**Age (years), median (i.q.r.)**	67 (55–76)	67 (55– 6)	69 (57– 77)	<0.001‡
< 50	2467 (17.6)	2448 (17.3)	1795 (14.8)	<0.001
50–64	3700 (26.5)	3696 (26.1)	3053 (25.1)	
65–79	5552 (39.7)	5562 (39.3)	4959 (40.8)	
≥ 80	2266 (16.2)	2451 (17.3)	2347 (19.3)	
**Sex ratio**	7248 : 6733	7203 : 6952	6497 : 5635	<0.001
Missing	4 (0.0)	2 (0.0)	22 (0.2)	
**BMI (kg/m^2^)**				<0.001
0–19	292 (2.1)	307 (2.2)	333 (2.7)	
20–24	2151 (15.4)	2170 (15.3)	2032 (16.7)	
25–30	2319 (16.6)	2292 (16.2)	2087 (17.2)	
> 30	3677 (26.3)	3586 (25.3)	2465 (20.3)	
Not registered*	5100 (36.5)	5452 (38.5)	4975 (40.9)	
Missing	438 (3.1)	342 (2.4)	256 (2.1)	
**Co-morbidities**				<0.001
Present	3956 (28.3)	6285 (44.4)	5495 (45.2)	
Not registered*	3741 (26.8)	2226 (15.7)	2138 (17.6)	
Missing	1878 (13.4)	196 (1.4)	150 (1.2)	
**Charlson Co-morbidity Index score**				<0.001
0–1	6107 (43.7)	6318 (44.6)	5065 (41.7)	
≥ 2	2259 (16.2)	3703 (26.2)	3244 (26.7)	
Not registered*	3766 (26.9)	3987(28.1)	3733 (30.7)	
Missing	1853 (13.2)	149 (1.1)	79 (0.6)	
**ASA fitness grade**				<0.001
I–II	6438 (46.0)	6210 (43.9)	5347 (44.0)	
III–V	5566 (39.8)	6123 (43.3)	5137 (42.3)	
Not registered*	1772 (12.7)	1761 (12.4)	1595 (13.1)	
Missing	209 (1.5)	63 (0.4)	75 (0.6)	
**Urgency of surgery**				
Acute setting	3577 (25.6)	3740 (26.4)	3579 (29.4)	<0.001
Elective setting	7230 (51.7)	6960 (49.2)	5427 (44.7)	<0.001
Unknown	1362 (9.7)	1496 (10.6)	1270 (10.4)	0.009
Not registered*	1816 (13.0)	1961 (13.9)	1878 (15.5)	
**Indication for surgery**				
Oncological	6260 (44.8)	6172 (43.6)	5712 (47.0)	<0.001
Non-oncological	7725 (55.2)	7985 (56.4)	6442 (53.0)	<0.001
**Perioperative SARS-CoV-2 infection**	—	—	75 (0.6)	
Confirmed before surgery	—	—	28 (0.2)	
Confirmed after surgery	—	—	62 (0.5)	
**Surgical audit**				
DATO—bariatric surgery	2661 (19.0)	2599 (18.4)	1543 (12.7)	<0.001
DCRA—colorectal surgery	2572 (18.4)	2355 (16.6)	1933 (15.9)	<0.001
DHBA—liver surgery	1190 (8.5)	1325 (9.4)	1215 (10.0)	<0.001
DHFA—hip fractures	1994 (14.3)	2226 (15.7)	2138 (17.6)	<0.001
DLCA—pulmonary surgery	2340 (16.7)	2444 (17.3)	2133 (17.5)	0.199
DSAA—aneurysm surgery	1772 (12.7)	1761 (12.4)	1595 (13.1)	0.245
DPCA—pancreatic surgery	626 (4.5)	636 (4.5)	663 (5.5)	<0.001
DUCA—Upper GI surgery	830 (5.9)	811 (5.7)	931 (7.7)	<0.001

Values are *n* (%) unless otherwise indicated.

* Variable not registered in certain surgery-specific audits. An overview of registered variables that are available in only a selection of audits is provided in *Table S1*. Non-registered data were excluded from analyses. An overview of patient characteristics for each surgical audit is presented in *Tables S2a–h*. DATO, Dutch Audit for Treatment of Obesity; DCRA, Dutch Colorectal Cancer Audit; DHBA, Dutch Hepatobiliairy Audit; DHFA, Dutch Hip Fracture Audit; DLCA-S, Dutch Lung Cancer Audit - Surgery; DSAA, Dutch Surgical Aneurysm Audit; DPCA, Dutch Pancreatic Cancer Audit; DUCA, Dutch Upper Gastro Intestinal Cancer Audit; GI, gastrointestinal. †Pearson’s χ^2^ test, except ‡Mann–Whitney *U* test.

### Procedures performed and treatment planning

**Fig. 1:**
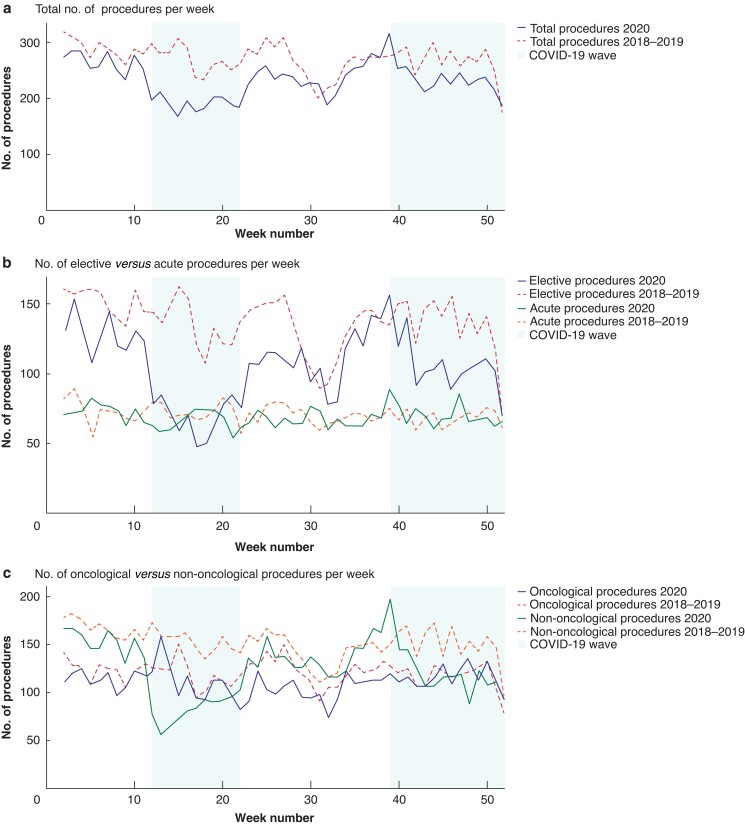
Numbers of patients who had surgery every week during 2020 compared with mean number of procedures performed in historical reference cohort of 2018–2019

During 2020, a total of 12 154 surgical procedures were carried out in the participating hospitals, representing a decrease of 14.1 and 13.1 per cent compared with 2019 and 2018 respectively. The largest decrease in the number of procedures performed in 2020 was observed during the first and second COVID-19 waves (29.2 and 12.2 per cent decrease respectively compared with equivalent period in 2018–2019 (*Fig. 1a*). A graphical representation of the procedures performed each week by audit is shown in *Fig. S2a–h*.

**a** Total number of procedures, **b** number of elective *versus* acute procedures, and **c** number of oncological *versus* non-oncological procedures undertaken per week.

### Procedures performed: elective *versus* acute surgery

Most of the procedures were done in an elective setting from 2018 to 2020. However, in 2020, a larger proportion of procedures was performed in an acute setting than in 2018 and 2019 (29.4 *versus* 25.6 and 26.4 per cent respectively; *P* < 0.001). A decrease in the number of elective procedures was observed during the first and second COVID-19 waves (49.7 and 23.2 per cent decrease compared with equivalent periods from the combined cohorts of 2018 and 2019 respectively; *P* < 0.001), whereas the absolute number of acute procedures remained stable (3579 in 2020, 3740 in 2019, and 3577 in 2018) (*Fig. 1b*).

### Procedures performed: oncological *versus* non-oncological surgical procedures

Although there was an 8.1 per cent decrease in the absolute number of oncological surgical procedures in 2020 compared with the combined cohort of 2018–2019 (5712 in 2020 *versus* 6216 in 2018–2019), the proportion of oncological surgical procedures performed in 2020 was higher than in the historical cohorts (47.0 per cent in 2020 *versus* 43.6 per cent in 2019 and 44.8 per cent in 2018; *P* < 0.001). The largest decrease, in absolute numbers, in oncological procedures occurred during the interim period (97 procedures per week in 2020 *versus* 115 in the participating hospitals in 2018–2019). Moreover, compared with oncological surgical care, a larger reduction in the number of non-oncological procedures was observed in 2020 *versus* the historical cohort of 2018–2019; there was an 18 per cent decrease in the absolute numbers (6442 procedures in 2020 *versus* 7855 in 2018–2019) and a reduced proportion of procedures (53.0 per cent in 2020 *versus* 56.4 per cent in 2019 and 55.2 per cent in 2018; *P* < 0.001). Notably, the largest differences were observed during the first (90 procedures per week in 2020 *versus* 161 in 2018–2019) and second (131 procedures per week in 2020 *versus* 154 in 2018–2019) COVID-19 waves (*Fig. 1c*). A graphical representation of the total number of surgical procedures performed in each audit is shown in *Fig. S2a–h*.

### Treatment planning: time to surgery and delay to surgery

In 2020, the median time to surgery was 28 (i.q.r. 2–104) days, which was shorter than the 34 (6–109) days in 2019 and 36 (8–120) days in 2018 (*P* < 0.001) (*Table 2*). The number of delayed surgical procedures reported in the additional COVID-19 survey was 839 (9.6 per cent), and the delay was most often due to reduced hospital capacity (717 of 839; 85.4 per cent). A delay to surgical procedures was more common for non-oncological than for oncological procedures (92 *versus* 8 per cent) (*Table 2*). For colorectal, upper gastrointestinal, and hepatobiliary procedures, the time to surgery was significantly shorter in 2020 than in the historical cohorts, whereas for pancreatic and bariatric procedures it was significantly longer (Tables  *S3a–h* and *Appendix S2*).

**Table 2:** Overview of surgical treatment planning for all included patients undergoing surgery between the 1 January 2018 and 31 December 2020

	2018 (*n* = 13 985)	2019 (*n* = 14 157)	2020 (*n* = 12 154)	*P*‡
**Time to surgery (days), median (i.q.r.)**				
Overall	36 (8–120)	34 (6–109)	28 (2–104)	<0.001§
Oncological surgery	33 (17–73)	33 (17–76)	33 (16–84)	0.483§
Non-oncological surgery	71 (1–169)	43 (1–144)	2 (1–160)	<0.001§
Not registered*	3106 (22.2)	3226 (22.8)	2837 (23.3)	
**Additional survey regarding delay to surgery**	–	–	*n* = 8709	
Surgical treatment postponed†	–	–	839 (9.6)	
Oncological	–	–	67 (0.8)	
Non-oncological	–	–	772 (8.9)	
Reason for delay†	–	–		
Capacity	–	–	717 (8.2)	
(Suspected) COVID-19 infection	–	–	53 (0.6)	
Other	–	–	47 (0.5)	
**Surgical treatment plan**				
Oncological	*n* = 6260	*n* = 6172	*n* = 5712	
Minimally invasive	3724 (59.5)	3803 (61.6)	3519 (61.1)	0.021
Open	1750 (28.0)	1547 (25.1)	1501 (26.0)	0.001
Local treatment	246 (3.9)	454 (7.4)	347 (6.1)	<0.001
Endovascular	0 (0)	0 (0)	0 (0)	1.000
Unknown	540 (8.6)	368 (6.0)	345 (6.0)	<0.001
Non-oncological	*n* = 7725	*n* = 7985	*n* = 6442	
Minimally invasive	4720 (61.1)	4876 (61.1)	3719 (57.7)	<0.001
Open	586 (7.6)	598 (7.5)	543 (8.4)	0.079
Local treatment	2 (0.0)	2 (0.0)	3 (0.0)	0.724
Endovascular	1214 (15.7)	1193 (14.9)	1093 (17.0)	0.007
Unknown	1202 (15.6)	1313 (16.4)	1084 (16.8)	0.105
**Additional survey regarding changes to surgical treatment plan**	–	–	*n* = 8700	
Surgical treatment plan changed†	–	–	149 (1.7)	
Reason for change in surgical approach	–	–		
Capacity	–	–	144 (1.7)	
COVID-19 infection	–	–	0 (0)	
Protection of team	–	–	1 (0.01)	
Other	–	–	1 (0.01)	
**Oncological neoadjuvant treatment plan**	*n* = 6260	*n* = 6172	*n* = 5712	
Received neoadjuvant treatment	1429 (23.0)	1392 (22.6)	1450 (25.6)	0.001
Chemotherapy	531 (8.8)	555 (9.1)	641 (11.3)	<0.001
Radiotherapy	170 (2.8)	115 (1.9)	105 (1.9)	0.001
Chemoradiotherapy	738 (12.3)	722 (11.8)	704 (12.4)	0.527
Missing	23 (0.4)	50 (0.8)	67 (1.2)	
**Additional survey regarding changes to neoadjuvant treatment plan**			*n* = 4428	
Neoadjuvant treatment plan changed†			26 (0.6)	
Other treatment			4 (0.1)	
Other scheme/dosing			6 (0.1)	
No therapy			11 (0.3)	
Other			5 (0.0)	

Values are *n* (%) unless otherwise indicated.

* Variable not registered in certain surgery-specific audits. An overview of registered variables that are available in only a selection of audits is provided in *Table S1*. Non-registered data were excluded from analyses. An overview of patient characteristics for each surgical audit is presented in *Tables S2a–h*. †As indicated in additional survey. ‡Pearson’s χ^2^ test, except §Mann–Whitney *U* test.

### Treatment planning: surgical and neoadjuvant treatment plans

Overall, minimally invasive techniques (such as laparoscopic or robot-assisted techniques) remained the dominant surgical approach. With regard to oncological surgical care, although it appears that a significant change in the use of minimally invasive surgical techniques was noted (61.1 per cent in 2020 *versus* 61.6 per cent in 2019 *versus* 59.5 per cent in 2018; *P* = 0.021), no change in the use of minimally invasive surgical techniques was noted when compared with the historical reference cohort together (61.1 per cent in 2020 *versus* 60.5 per cent in 2018–2019; *P* = 0.179). In 1.7 per cent of the surgical procedures undertaken in 2020 (149 patients), a change in the surgical approach was reported in the additional COVID-19 survey. The main reason for the change was lack of capacity (96.6 per cent). Regarding neoadjuvant treatment plans, a change in the neoadjuvant treatment plan was reported for 26 patients (0.6 per cent), mainly leading to the cancellation of neoadjuvant treatment (11 of 26) (*Table 2*).

### Surgical outcomes in 2020

#### Duration of hospital stay

Median duration of hospital stay for oncological procedures was significantly shorter during 2020 than in the historical reference group: 5 (i.q.r. 3–9) *versus* 6 (4–10 days) respectively (*P* < 0.001) (*Table 3*). With regard to the different time intervals in 2020 compared with 2018–2019, this reduction in duration of hospital stay for oncological procedures occurred during both the COVID-19 waves and the interim period (first wave: 5 *versus* 6 days respectively; second wave: 5 *versus* 6 days; interim period: 5 *versus* 6 days) (*Table 4*). A significant audit-specific decrease in duration of hospital stay compared with the historical cohort was observed in one or more intervals in 2020 for patients who underwent colorectal, hepatobiliary, pulmonary, upper gastrointestinal, aneurysm, and hip fracture procedures. Audit-specific data on postoperative surgical outcomes are available in *Tables S4a–h* and *Appendix S2*.

**Table 3:** Overview of postoperative surgical outcomes for all included patients undergoing surgery in 2020 compared with historical reference cohort (2018–2019)

	2018–2019 (*n* = 28 142)	2020 (*n* = 12 154)	*P*§
**Duration of hospital stay (days), median i.q.r.)**	4 (2–8)	4 (2–8)	0.064¶
Oncological	6 (4–10)	5 (3–9)	<0.001¶
Non-oncological	2 (1–6)	3 (1–6)	<0.001¶
**Severe complication** *	2346 (8.3)	1052 (8.7)	0.021
Oncological	2031 (16.4)	912 (16.0)	0.498
Non-oncological	315 (2.0)	140 (2.2)	0.08
Not registered†	7753 (27.5)	3733 (30.7)	
Missing	45 (0.2)	9 (0.1)	
**Readmission within 30 days**	1659 (5.9)	751 (6.2)	0.448
Oncological	1165 (9.4)	558 (9.8)	0.424
Non-oncological	494 (3.0)	193 (3.1)	0.728
Not-registered†	5410 (19.2)	2138 (17.6)	
Missing	183 (0.7)	111 (0.9)	
**Postoperative death**	819 (2.9)	373 (3.1)	0.373
Oncological	298 (2.4)	150 (2.7)	0.063
Non-oncological	521 (3.4)	212 (3.3)	0.894
COVID-19‡		11 (0.1)	
**ICU admission**	4733 (16.8)	1999 (16.5)	<0.001
Oncological	2698 (21.7)	1106 (19.4)	<0.001
Non-oncological	2035 (13.0)	893 (13.9)	<0.001
Not registered†	4220 (15.0)	2138 (17.5)	
Missing	1082 (3.8)	188 (1.5)	
**Duration of ICU stay (days), median (i.q.r.)**	0 (0–1)	0 (0–1)	<0.001¶
**Oncological data**	*n* = 12 432	*n* = 5712	
**Resection margin status**			<0.001
R0	9356 (75.3)	4171 (73.0)	
R1	624 (5.0)	368 (6.4)	
R2	466 (3.7)	175 (3.1)	
Unknown	708 (5.7)	353 (6.2)	
Non-malignant disease	375 (6.2)	203 (3.6)	
Not-registered†	862 (6.9)	420 (7.2)	
Missing	466 (3.7)	175 (3.1)	
**Disease stage**			<0.001
0	306 (2.5)	138 (2.4)	
I	2629 (21.1)	1087 (19.0)	
II	2437 (19.6)	1193 (20.9)	
III	2613 (21.0)	1163 (20.4)	
IV	407 (3.3)	177 (3.1)	
X	174 (1.4)	122 (2.1)	
Non-malignant disease	642 (5.2)	353 (6.2)	
Not registered†	3461 (19.1)	1127 (19.7)	
Missing	352 (1.9)	32 (0.6)	

Values are *n* (%) unless otherwise indicated.

* Needing reintervention, ICU admission and/or causing death. †Variable not registered in certain surgery-specific audits. An overview of registered variables that are available in only a selection of audits is provided in *Table S1*. Non-registered data were excluded from analyses. ‡*P*atients with a confirmed perioperative COVID-19 infection who died after surgery from any cause (including non-COVID related causes). §Pearson’s χ^2^ test, except ¶Mann–Whitney *U* test.

**Table 4:** Overview of postoperative surgical outcomes during first COVID-19 wave, second COVID-19 wave, and interim period in 2020 compared with those of historical reference cohorts that underwent surgery between 1 January 2018 and 31 December 2020

	First wave (*n* = 1919)	Reference first wave (*n* = 5430)	*P*§	Second wave (*n* = 3310)	Reference second wave (*n* = 7537)	*P*§	Interim period (*n* = 4021)	Reference interim period (*n* = 8940)	*P*§
**Duration of hospital stay (days), median (i.q.r.)**	5 (3–8)	4 (2–8)	<0.001	4 (2–8)	4 (2–8)	0.060	4 (2–7)	4 (2–8)	0.003
Oncological	5 (3–9)	6 (4–9)	0.046	5 (3–9)	6 (3–10)	0.011	5 (3–9)	6 (4–10)	0.001
Non-oncological	4 (2–7)	3 (1–7)	<0.001	3 (1–7)	2 (1–6)	0.008	3 (1–6)	2 (1–6)	0.588
**Severe complication** *	187 (9.7)	458 (8.4)	<0.001	300 (9.1)	589 (7.8)	0.002	337 (8.4)	795 (8.9)	0.778
Oncological	177 (15.8)	393 (16.6)	0.574	258 (15.9)	505 (15.2)	0.558	285 (16.4)	704 (17.1)	0.512
Non-oncological	10 (1.3)	65 (2.1)	0.707	42 (2.5)	84 (2.0)	0.006	52(2.3)	91 (1.9)	0.178
Not registered†	601 (31.1)	1525 (28,1)		1047 (31.6)	2035 (27.0)		1216 (30.2)	2437 (27.3)	
Missing	3 (0.2)	8 (0.1)		1 (0.0)	13 (0.2)		2 (0.0)	17 (0.2)	
**Readmission within 30 days**	135 (7.0)	340 (6.3)	0.222	199 (6.0)	431 (5.7)	0.693	266 (6.6)	535 (6.0)	0.198
Oncological	110 (9.8)	240 (10.1)	0.335	155 (9.6)	295 (8.9)	1.000	193 (11.1)	394 (9.6)	0.401
Non-oncological	25 (3.1)	100 (3.3)	0.260	44 (2.6)	136 (3.2)	0.524	73 (3.2)	141 (2.9)	0.366
Not registered†	380 (19.8)	1063 (19.6)		566 (17.1)	1440 (19.1)		715 (17.8)	1692 (18.9)	
Missing	18 (0.9)	29 (0.5)		60 (1.8)	69 (0.9)		20 (0.5)	43 (0.5)	
**Postoperative death**	57 (3.0)	164 (3.0)	0.342	136 (4.1)	227 (3.0)	0.011	103 (2.6)	227 (2.5)	0.928
Oncological	19 (1.9)	63 (2.7)	0.308	68 (4.4)	88 (2.6)	0.004	39 (2.2)	76 (1.8)	0.539
Non-oncological	36 (4.5)	101 (3.3)	0.089	60 (3.8)	139 (3.3)	0.460	63 (2.8)	151 (3.1)	0.551
Perioperative COVID-19 infection‡	2 (0.1)	–		8 (0.2)	–		1 (0.0)	–	
**ICU admission**	354 (18.4)	918 (16.9)	0.030	530 (16.0)	1246 (16.5)	<0.001	618 (15.4)	1516 (17.0)	<0.001
Oncological	223 (19.9)	524 (22.1)	0.062	291 (18.0)	697 (21.0)	<0.001	332 (19.1)	989 (21.8)	<0.001
Non-oncological	131 (16.4)	394 (12.9)	<0.001	239 (14.1)	549 (13.0)	<0.001	286 (12.5)	618 (12.8)	<0.001
Not registered†	380 (19.8)	840 (15.5)		566 (17.1)	1115 (14.8)		715 (17.8)	1310 (14.7)	
Missing	54 (2.8)	190 (3.5)		39 (1.2)	268 (3.6)		58 (1.4)	398 (4.5)	
**Duration of ICU stay (days), median (i.q.r.)**	0 (0–1)	0 (0–1)	0.076	0 (0–1)	0 (0–1)	0.001	0 (0–1)	0 (0–1)	0.064
**Oncological data**	*n* = 821	*n* = 1750		*n* = 1204	*n* = 2413		*n* = 1253	*n* = 3068	
**Resection margins**			0.291			0.001			0.164
R0	695 (84.7)	1495 (85.4)		1011 (84.0)	2097 (86.9)		1053 (84.0)	2618 (85.3)	
R1	53 (6.5)	85 (4.9)		77 (6.4)	104 (4.3)		72 (5.7)	139 (4.5)	
R2	3 (0.4)	2 (0.1)		4 (0.3)	7 (0.3)		5 (0.4)	6 (0.2)	
Unknown	39 (3.5)	80 (3.4)		52 (4.3)	124 (5.1)		50 (3.9)	135 (4.4)	
Not registered†	33 (2.9)	78 (3.3)		63 (5.2)	70 (2.9)		68 (5.4)	136 (4.4)	
Missing	79 (7.0)	176 (7.4)		112 (11.1)	237 (11.3)		137 (10.9)	270 (8.8)	
**Disease stage**			0.191			<0.001			<0.001
0	42 (5.1)	60 (3.4)		38 (3.2)	80 (3.3)		32 (2.6)	101 (3.3)	
I	220 (26.8)	501 (28.6)		309 (25.7)	708 (29.3)		312 (24.9)	919 (30.0)	
II	234 (28.5)	438 (25.0)		324 (26.9)	657 (27.2)		356 (28.4)	831 (27.1)	
III	212 (25.8)	509 (29.1)		349 (29.0)	698 (28.9)		348 (27.8)	842 (27.4)	
IV	35 (4.3)	84 (4.8)		42 (3.5)	121 (5.0)		68 (5.4)	119 (3.9)	
X	20 (2.4)	38 (2.2)		42 (3.5)	28 (1.2)		36 (2.9)	60 (2.0)	
**Not registered†**	234 (20.9)	439 (18.5)		300 (24.8)	646 (26.7)		361 (28.8)	752 (28.7)	
Missing	58 (7.1)	120 (6.9)		100 (8.3)	121 (5.0)		101 (8.1)	196 (6.4)	

Values are *n* (%) unless otherwise indicated.

* Needing reintervention, ICU admission and/or causing death. †Variable not registered in certain surgery-specific audits. An overview of registered variables that are available in only a selection of audits is provided in *Table S1*. Non-registered data were excluded from analyses. An overview of postoperative surgical outcomes for each audit is available in *Table S4*.

‡ *P*atients with a confirmed perioperative COVID-19 infection who died after surgery from any cause (including non-COVID related causes). §Pearson’s χ^2^ test, except ¶Mann–Whitney *U* test.

#### Complication and readmission rates

For the cohort for which complications, reinterventions, ICU admissions, and mortality were registered (*Table S1a*,*b* and *Appendix S2*), severe complications (defined as need for reintervention, ICU admission and/or causing death) were documented more frequently during 2020 than for the historical reference cohort (8.7 *versus* 8.3 per cent; *P* = 0.021). This increase in complication rate was present during both COVID-19 waves in 2020 compared with historical reference group (first wave: 9.7 *versus* 8.4 per cent, *P* < 0.001; second wave: 9.1 *versus* 7.9 per cent, *P* = 0.002) (*Table 4*). However, when surgery-specific audit data were reviewed, a significant difference in severe complication rate was not observed for oncological surgical care or for any of the individual audits (*Tables S4a–h* and *Appendix S2*).

For both oncological and non-oncological procedures, rates of readmission within 30 days in 2020 were similar to those in 2018–2019 (6.2 *versus* 5.9 per cent; *P* = 0.448) (*Table 3*). However, the audit-specific data showed a significantly higher rate of readmissions within 30 days for upper gastrointestinal procedures during the second COVID-19 wave (*Tables S4a–h* and *Appendix S2*).

#### ICU stay and mortality rates

During 2020, the proportion of ICU admissions decreased significantly compared with that in the historical cohort (16.5 *versus* 16.8 per cent; *P* < 0.001). During the first COVID-19 wave, a higher percentage of ICU admissions was observed than in the historical reference cohort (18.4 *versus* 16.9 per cent; *P* = 0.030), in contrast to the second COVID-19 wave and the interim period, during which there was a lower proportion of ICU admissions (second wave: 16.0 *versus* 16.5 per cent, *P* < 0.001; interim period: 15.4 *versus* 17.0 per cent, *P* < 0.001) (*Table 4*). Audit-specific ICU admission rates were also found to decrease for colorectal, pulmonary, upper gastrointestinal, and abdominal aneurysm procedures (*Tables S4a–h* and *Appendix S2*). Median duration of ICU stay was shorter in 2020 than in 2018–2019 in the specific audits of pulmonary, upper gastrointestinal, and abdominal aneurysm procedures.

During 2020, postoperative mortality rates remained unchanged compared with those in the historical reference cohort (3.1 per cent in 2020 and 2.9 per cent in 2018–2019; *P* = 0.373). In total, 11 surgical patients (0.1 per cent) in this study cohort died after surgery because of a COVID-19 infection. Postoperative mortality rates increased during the second COVID-19 wave for the total cohort (4.1 *versus* 3.0 per cent; *P* = 0.011). The highest rate of COVID-19-positive mortality was observed during the second COVID-19 wave (8 patients). For patients who underwent colorectal surgery, increased audit-specific mortality rates were documented during the second COVID-19 wave (8.2 *versus* 3.7 per cent; *P* < 0.001).

## Discussion

The total number of surgical procedures decreased significantly in 2020 compared with 2018–2019; reductions in surgical volume were most substantial during the first and second COVID-19 waves. In particular, the number of elective procedures decreased during these intervals. However, the weekly numbers of acute surgical procedures remained stable. Moreover, the proportion of surgical oncological procedures increased in 2020 compared with the historical cohort of 2018–2019, even during both COVID-19 waves. The overall time to surgery decreased in 2020, except for pancreatic and bariatric surgical care, for which there was a longer waiting time. For almost 10 per cent of all surgical procedures undertaken, surgeons stated that the delay to surgery was a result of reduced capacity. For oncological surgical procedures, the duration of hospital stay decreased in 2020, whereas the complication and readmission rates did not differ between 2020 and the historical reference cohorts.

This is the first nationwide surgical study involving prospective collection and analysis of consecutive data from patients who were treated surgically in 50 participating hospitals, covering eight surgical audits in the Netherlands. Such consecutive data allowed the investigators to detect trends throughout 2020, and to subsequently compare these with historical cohorts. The present study was undertaken in the Netherlands, a country where surgical care is standardized. Therefore, planning, organization, and the outcomes of surgical care are comparable between hospitals, which is an important advantage of this study compared with other large studies of surgery and COVID-19, such as those carried out by the GlobalSurg COVIDSurg Network^5,11^. By including hospitals from various countries, encompassing low-, middle- and high-income countries with wide heterogeneity in surgical care, the GlobalSurg COVIDSurg Network inevitably included more varied and less standardized and comparable surgical care^12–14^. Furthermore, many of the published surgical studies on COVID-19 primarily focused on the postoperative outcomes of COVID-19-positive patients undergoing surgery^12,13,15^ or solely on complex surgical care^16–20^, whereas the present study focused on a broader spectrum of surgical procedures in the Netherlands. It also covers the whole of the year 2020, including both the first and the second COVID-19 waves, whereas other studies^5,12–18^ have reviewed smaller time intervals ranging from 1 to 6 months, or used prediction modelling without actual patient data.

The present results have shown that, although the total number and proportion of elective surgical procedures decreased during both COVID-19 waves, the proportion of oncological surgical procedures increased. In line with the results of the Dutch joint quality registries report, a reduction in the number of oncological colorectal procedures was noted. This decrease in number of colorectal procedures could be explained by the temporary cancellation of the national screening programme for colorectal cancer during the first COVID-19 wave^4,21,22^. Another factor possibly leading to delay in diagnosis of colorectal, upper gastrointestinal, and hepatobiliary diseases may have been the limited access to endoscopy units during intervals with peak incidence of COVID-19^21,22^. However, as this study focused on surgical patterns of care and time to surgery was calculated based on the first appointment with the surgeon, this delay in diagnosis is not reflected here. The Dutch joint quality registries report^21^ described an 11 per cent decrease in the number of oncological procedures performed in 2020, which is considered a greater reduction than found in the present study. This discrepancy may be attributed to the voluntary nature of the present study.

The reduction in elective surgical care in this study is consistent with the findings of an analysis by the Global COVIDSurg Collaborative^5^, in which a model was used to predict the percentage downscaling of elective procedures during the 12 weeks of peak COVID-19 disruption. This model predicted a 72.3 per cent cancellation rate, predominantly affecting benign diseases (81.7 per cent) as opposed to procedures for malignant diseases (37.7 per cent). These findings, predominantly indicating the cancellation of non-oncological elective healthcare during the first COVID-19 wave, are in line with the present results and some other recent studies, which described a decrease in procedures performed^19^, especially in March–April 2020^23,24^. One of these, a neurosurgical study^23^ undertaken in a large centre, also reported an increase in procedures performed in July 2020, with a decrease in operating capacity of between 30 and 55 per cent. The authors found that emergency procedures were not affected by decreased operating room capacity, which is in line with the results of the present study. Moreover, the fact that oncological surgical care seemed to be less affected by the COVID-19 pandemic is supported by the findings of Foo *et al*.^25^, who noted that, even with precautionary measures, oncological colorectal procedures were still performed according to the standard of care in three Asian hospitals. Rottoli *et al*.^20^, who looked at stage migration and worse postoperative outcomes after colorectal surgery owing to COVID-19 in 20 hospitals in northern Italy in 2020 compared to 2019, found that undergoing surgery in 2020 was not a predictor of advanced oncological stage and more surgical complications.

Concerning the impact of COVID-19 on duration of postoperative hospital stay, the published literature remains ambiguous, with a shorter stay reported after hip fracture and colorectal cancer surgery^26,27^, and a longer stay after elective orthopaedic hip surgery^28^. Shorter hospital stay might be explained by the endeavour of healthcare personnel to discharge patients earlier than usual, to minimize the risk of admission-related COVID-19 infections, or to achieve a higher hospital capacity because of staffing problems and fear of potentially high volumes of patients with COVID-19 being expected to arrive^29,30^. Another factor that may have influenced duration of hospital stay after hip fracture repairs could have been the institutional outflow mechanisms to rehabilitation facilities. At first, transferring patients to other healthcare facilities after surgery was more cumbersome as a result of mandatory testing policies and limited access owing to COVID-19 outbreaks in rehabilitation facilities^4^. However, high COVID-related mortality rates among people aged 60 years and above, and faster outflow to rehabilitation facilities or nursing homes could have led to a shorter hospital stay during later stages of the COVID-19 pandemic^4^. There was no evidence in the present study to suggest that more rapid discharge from hospital was associated with higher complication and readmission rates.

This study has certain limitations. One limitation concerns the variability in registration of data by the different audits. Differences exist in the way variables are registered, which results in missing data for variables that were not registered in specific audits. For example, not all audits register reinterventions or ICU admissions, or use the Clavien–Dindo classification for complications. Second, overall data from combined audits should always be interpreted with caution, as they cover a broad spectrum of diverse procedures with their own indications and treatment patterns. These general data and the overall trends deduced from them might not do justice to audit-specific trends that could be present. For example, no information was available about changes in planned anastomoses/stoma formation as a result of the COVID-19 pandemic. Furthermore, increased time to pancreatic surgery could, for example, be explained by alterations in neoadjuvant treatment strategies advised in guidelines^31^, regardless of the COVID-19 pandemic. Another limitation lies in the fact that only patients who underwent surgery in 2018–2020 were registered, and so conclusions could not be drawn regarding those who did not receive surgical care. Changes in surgical therapy plans for procedures that were postponed from 2020 to 2021 or surgical procedures that were cancelled because of COVID-19 were not captured here. Furthermore, because this study only included data from patients who underwent surgery up to December 2020, no assessment of long-term consequences, such as increased waiting lists or possible stage migration, was possible. Finally, for logistical reasons, inclusion for this study was started on 1 January 2020. However, the first COVID wave started later that year and the first patients would not have been affected by the pandemic. This was addressed by showing figures for the COVID waves and comparing these with historical data, not only using data for the whole year.

From a clinical perspective, the decrease in number of non-oncological procedures compared with the number of elective oncological and acute procedures reflects the general thought that the most urgent healthcare was prioritized and that, by postponing less urgent surgical care, capacity was preserved to maintain oncological and acute surgical care^32^. Another possible explanation for the relatively small decline in most urgent surgical care might be the observed shortening of hospital stay, which potentially led to a higher hospital capacity as a result of a higher turnover rate.

The findings of this study suggest that the aftermath of the pandemic, reflected by increased waiting lists, will soon become apparent for patients undergoing elective non-oncological surgery. With the current shortage of ICU capacity and trained staff, the observed decrease in ICU admissions for oncological procedures may represent an important lead to reinvestigate the indications for standardized postoperative ICU admission for each procedure.

Although the downscaling of healthcare was inevitable, it did not result in an increased volume of acute surgical care compared with the pre-COVID-19 era. Moreover, the decline in healthcare did not lead to dismal surgical outcomes in patients who did undergo surgery, because the duration of hospital stay and ICU admission rates decreased, and, most importantly, there was no increase in complication or mortality rates. However, from a patient perspective, the aftermath of the pandemic in terms of increased waiting lists and insecurity felt will probably become apparent in the coming years. Nonetheless, although this has been a tour de force for all healthcare workers and patients whose operations were cancelled or delayed, this study has shown the potential for even more efficient surgical care, which is greatly needed in order to catch up with all postponed surgical care.

## Collaborators

Dutch CovidSurg Collaborative Study Group: J.N. Helleman, C.L. Koningswoud-Terhoeve (Admiraal De Ruyter Hospital), E. Belt, J.A.B. van der Hoeven, G.M.H. Marres, F. Tozzi, E.M. von Meyenfeldt (Albert Schweitzer Hospital, Dordrecht, The Netherlands). R.R.J. Coebergh - van den Braak, S. Huisman, A.M. Rijken (Amphia hospital, Breda, the Netherlands), R. Balm, F. Daams, C. Dickhoff, W.J. Eshuis, S.S. Gisbertz, H.R. Zandbergen (Amsterdam UMC, Amsterdam, the Netherlands), K.J. Hartemink, S.A. Keessen, N.F.M. Kok, K.F.D. Kuhlmann, J.W. van Sandick, A.A. Veenhof. A. Wals (Antoni van Leeuwenhoek Hospital - Nederlands Kanker Instituut (AvL-NKI), Amsterdam, the Netherlands), M.S. van Diepen, L. Schoonderwoerd, C.T. Stevens (Bernhoven Hospital, Uden, the Netherlands), D. Susa (Bravis Hospital), B.L.W. Bendermacher, N. Olofsen (Canisius-Wilhelmina Hospital, Nijmegen, the Netherlands), M. van Himbeeck, I.H.J.T. de Hingh, H.J.B. Janssen, M.D.P. Luyer, G.A.P. Nieuwenhuijzen, M. Ramaekers, R. Stacie (Catharina Hospital, Eindhoven, the Netherlands), A.K. Talsma, M.W. Tissink (Deventer Hospital, Deventer, the Netherlands), D. Dolmans (Diakonessenhuis Utrecht, the Netherlands), R. Berendsen, J. Heisterkamp, W.A. Jansen, M. de Kort-van Oudheusden, R.M. Matthijsen (Elisabeth Tweesteden Hospital, Eindhoven, the Netherlands), D.J. Grünhagen, S.M. Lagarde, A.P.W.M. Maat, P.C. van der Sluis, R.B. Waalboer (Erasmus Medical centre, Rotterdam, the Netherlands), V. Brehm, J.P. van Brussel, M. Morak (Franciscus Gasthuis & Vlietland, Rotterdam, the Netherands), E.D. Ponfoort, J.E.M. Sybrandy (Gelderse Vallei, Ede, the Netherlands), P.L. Klemm, W. Lastdrager, H.W. Palamba (Gelre Ziekenhuizen, Apeldoorn, the Netherlands), S.M. van Aalten, L.N.L. Tseng (Groene Hart Hospital, Gouda, the Netherlands), K.E.A. van der Bogt, W.J. de Jong, J.W.A Oosterhuis, Q. Tummers, G.M. van der Wilden (Haaglanden Medical centre), S. Ooms, E.H. Pasveer, H.T.C. Veger (Haga Hospital, Den Haag, the Netherlands), M.J. Molegraafb, V.B. Nieuwenhuijs, G.A. Patijn, M.E.V. van der Veldt (Isala Klinieken, Zwolle, the Netherlans), D. Boersma, S.T.W. van Haelst, I.D. van Koeverden, M.L. Rots (Jeroen Bosch Hospital, Den Bosch, the Netherlands), B.A. Bonsing, N. Michiels, O.D. Bijlstra, J. Braun, D. Broekhuis, H.W. Brummelaar, H.H. Hartgrink, A. Metselaar, J.S.D Mieog, I.B. Schipper, W.O. de Steur, (Leids University Medical centre, Leiden, the Netherlands), B. Fioole, E.C. Terlouw (Maasstad hospital, Rotterdam, the Netherlands), C. Biesmans, J.W.A.M. Bosmans, S.A.W. Bouwense, S.H.E.M. Clermonts, M.M.E. Coolsen, B.M.E. Mees, G.W.H. Schurink (Maastricht University Medical centre, Maastricht, the Netherlands), J.W. Duijff, T. van Gent, L.C.F. de Nes, D. Toonen (Maas hospital, Beugen, the Netherlands), M.J. Beverwijk, E. van den Hoed, B. Keizers, W. Kelder, B.P.J.A Keller, B.B. Pultrum, E. van Rosum, A.G. Wijma (Martini Hospital, Groningen, the Netherlands), F. van den Broek, W.K.G. Leclercq, M.J.A. Loos, J.M.L. Sijmons, R.H.D. Vaes, P.J. Vancoillie (Maxima Medical centre, Veldhoven, the Netherlands), E.C.J. Consten, J.M.J. Jongen, P.M. Verheijen, V. van Weel (Meander Medical centre, Amersfoort, the Netherlands), C.H.P. Arts, J. Jonker, G. Murrmann-Boonstra, J.P.E.N. Pierie, J. Swart (Medical centre Leeuwarden, Leeuwarden, the Netherlands), E.B. van Duyn, R.H. Geelkerken, R. de Groot, N.L. Moekotte, A. Stam, A. Voshaar (Medisch Spectrum Twente, Enschede, the Netherlands), G.J.D. van Acker, R.M.A. Bulder, D.J. Swank (Dutch Obesity Clinic, Den Haag, the Netherlands), I.T.A. Pereboom (Nij Smellinghe hospital, Drachten, the Netherlands), W.H. Hoffmann, M. Orsini (Reinier De Graaf, Delft, the Netherlands), J.J. Blok, J.H.P. Lardenoije, M.M.P.J. Reijne, P. van Schaik, L. Smeets, S.M.M. van Sterkenburg (Rijnstate Hospital, Arnhem, the Netherlands), N.J. Harlaar, S. Mekke, T. Verhaakt (Rode Kruis Hospital, the Netherlands), E. Cancrinus (Sint Anna Hospital, Geldrop, the Netherlands), G.W. van Lammeren, I.Q. Molenaar, H.C. van Santvoort, A.W.F. Vos (Sint Antonius Hospital, Nieuwegein, the Netherlands), A.P. Schouten- van der Velden, K. Woensdregt (Sint Jansdal Hospital, Hardewijk, the Netherlands), S.P. Mooy-Vermaat, D.M. Scharn (Slingeland Hospital, Doetinchem, the Netherlands), H.A. Marsman, F. Rassam (Onze Lieve Vrouwe Gasthuis, Amsterdam, the Netherlands), F.R. Halfwerk (Thoraxcentrum Twente, Medisch Spectrum Twente, Enschede, the Netherlands), A.J. Andela, C.I. Buis, G.M. van Dam, K. ten Duis, B. van Etten, L. Lases, M. Meerdink, V.E. de Meijer, B. Pranger, S. Ruiter, M. Rurenga, A. Wiersma, A.R. Wijsmuller (University Medical Centre Groningen, Groningen, the Netherlands), K.I. Albers, P.B. van den Boezem, B. Klarenbeek, B.M. van der Kolk, C.J.H.M. van Laarhoven, E. Matthée, N. Peters, C. Rosman, A.M.A. Schroen, M.W.J. Stommel, A.F.T.M. Verhagen, R. van der Vijver, M.C. Warlé, J.H.W. de Wilt (University Medical centre St. Radboud, Nijmegen, the Netherlands), J.W. van den Berg, T. Bloemert, G.J. de Borst, E.H. van Hattum, C.E.V.B. Hazenberg, J.A. van Herwaarden, R. van Hillegerberg, T.E. Kroese, B.J. Petri, R.J. Toorop (University Medical centre Utrecht, Utrecht, the Netherlands), F. Aarts, R.J.L. Janssen, S.H.P. Janssen-Maessen, M. Kool, H. Verberght (VieCuri Medical centre, Venlo, the Netherlands), D.E. Moes, J.W. Smit, A.M. Wiersema, Westfriesgasthuis / Dijklander hospital, Hoorn, the Netherlands), B.P. Vierhout, B. de Vos (Wilhelmina Hospital, Assen, the Netherlands), F.C. den Boer, N.A.M. Dekker (Zaans Medical centre, Zaandam, the Netherlands), J.M.J. Botman, M.J. van Det, E.C. Folbert, E. de Jong, J.C. Koenen, E.A. Kouwenhoven, I. Masselink, L.H. Navis (ziekenhuisgroep Twente, Hengelo, the Netherlands), H.J. Belgers, M.N. Sosef, J.H.M.B. Stoot (Zuyderland Medical centre, Heerlen, the Netherlands).

Dutch CovidSurg Collaborative study group: see *Appendix S4 and S5*.

## Supplementary Material

znac301_Supplementary_Data

## Data sharing

Anonymized individual-participant data will be made available upon request to the corresponding authors after the date of publication, with approval of the operations and dissemination committees within DICA, as well as the completion of a data-sharing agreement.
