# Erratum to: Impact of the COVID-19 pandemic on surgical care in the Netherlands

**DOI:** 10.1093/bjs/znac428

**Published:** 2022-11-17

This is an erratum to: Michelle R de Graaff, Rianne N M Hogenbirk, Yester F Janssen, Arthur K E Elfrink, Ronald S L Liem, Simon W Nienhuijs, Jean Paul P M de Vries, Jan Willem Elshof, Emiel Verdaasdonk, Jarno Melenhorst, H L van Westreenen, Marc G H Besselink, Jelle P Ruurda, Mark I van Berge Henegouwen, Joost M Klaase, Marcel den Dulk, Mark van Heijl, Johannes H Hegeman, Jerry Braun, Daan M Voeten, Franka S Würdemann, Anne Loes K Warps, Anna J Alberga, J Annelie Suurmeijer, Erman O Akpinar, Nienke Wolfhagen, Anne Loes van den Boom, Marieke J Bolster-van Eenennaam, Peter van Duijvendijk, David J Heineman, Michel W J M Wouters, Schelto Kruijff, the Dutch CovidSurg Collaborative Study Group, Impact of the COVID-19 pandemic on surgical care in the Netherlands, *British Journal of Surgery*, 2022; znac301, https://doi.org/10.1093/bjs/znac301

In the originally published version of this article, Rianne N M Hogenbirk was erroneously not recognized as one of the first authors.

This error has been corrected.

