# Supplementary material for: Impact of the COVID-19 pandemic on surgical care in the Netherlands
Source: Br J Surg. 2022 Sep 6;109(12):1282–92. doi: 10.1093/bjs/znac301 (PMC10364688; doi:10.1093/bjs/znac301)
Supplement: znac301_Supplementary_Data [file znac301_supplementary_data.docx]

**Supplementary Materials**

**Appendix S1: COVIDSurg Collaborative Study Group**

**Appendix S2: Supplementary Tables**

1. Overview of registered variables per audit
2. Details of audit-specific demographics are presented in Tables S2A–S2H.
3. Details of audit-specific surgical patterns of care are presented in Tables S3A–S3H.
4. Details of audit-specific surgical outcomes during the first and second COVID-19 waves and the interim period of 2020

**Appendix S3: Supplementary Figures**

1. Figure S1: Flowchart of inclusion of participating hospitals and operated patients per surgical audit in the Dutch COVIDSurg II Snapshot Study
2. Figures S2A–S2H: The number of procedures performed per audit per week.

**Appendix S4: Case Report Form COVIDSurg snapshot study**

**Appendix S5: Collaborating Centres per Audit**

**Appendix S1: Collaborators COVIDsurg II Snapshot Study**

List of collaborators of the DutchCOVIDSurg II Snapshot Study

| **Affiliation** | **Last name** | **Initials** | **Academic titles** |
| --- | --- | --- | --- |
| Groene Hart Ziekenhuis | Aalten, van | S.M. | MD, PhD |
| VieCuri Medisch Centrum | Aarts | F | MD, PhD |
| Nederlandse Obesitas Kliniek West, Den Haag | Acker, van | G.J.D. | MD, PhD |
| Universitair Medisch Centrum St. Radboud | Albers | K.I. | MD |
| Universitair Medisch Centrum Groningen (UMCG) | Andela | A.J. | MD |
| Medisch Centrum Leeuwarden | Arts | C.H.P. | MD, PhD |
| Amsterdam UMC | Balm | R | MD, PhD |
| Zuyderland Medisch centrum | Belgers | H.J. | MD, PhD |
| Albert Schweitzer Ziekenhuis | Belt | E | MD, PhD |
| Canisius-Wilhelmina Ziekenhuis | Bendermacher | B.L.W. | MD, PhD |
| Elisabeth Tweesteden Ziekenhuis | Berendsen | R | MSc |
| Universitair Medisch Centrum Utrecht | Berg, van den | J.W. | MD, PhD |
| Martini Ziekenhuis | Beverwijk | M.J. |  |
| Maastricht Universitair Medisch Centrum | Biesmans | C | BSc. |
| Leids Universitair Medisch Centrum (LUMC) | Bijlstra | O.D. | MD |
| Universitair Medisch Centrum Utrecht | Bloemert | T. | MSc. |
| Rijnstate Ziekenhuis | Blok | J.J. | MD, PhD |
| Zaans Medisch Centrum | Boer, den | F.C. | MD, PhD |
| Jeroen Bosch Ziekenhuis | Boersma | D | MD, PhD |
| Universitair Medisch Centrum St. Radboud | Boezem, van den | P.B. | MD, PhD |
| Haaglanden Medisch Centrum | Bogt, van der | K.E.A. | MD, PhD |
| Leids Universitair Medisch Centrum | Bonsing | B.A. | MD, PhD |
| Universitair Medisch Centrum Utrecht | Borst, de | G.J. | MD, PhD |
| Maastricht Universitair Medisch Centrum | Bosmans | J.W.A.M. | MD, PhD |
| Ziekenhuisgroep Twente | Botman | J.M.J. | MD |
| Maastricht Universitair Medisch Centrum | Bouwense | S.A.W. | MD, PhD |
| Leids Universitair Medisch Centrum (LUMC) | Braun | J | MD, PhD |
| Franciscus Gasthuis & Vlietland | Brehm | V | MD |
| Maxima Medisch Centrum | Broek, van den | F | MD |
| Leids Universitair Medisch Centrum (LUMC) | Broekhuis | D | MD |
| Leids Universitair Medisch Centrum (LUMC) | Brummelaar | H.W. | MD |
| Franciscus Gasthuis & Vlietland | Brussel, van | J.P. | MD, PhD |
| Universitair Medisch Centrum Groningen (UMCG) | Buis | C | MD, PhD |
| Nederlandse Obesitas Kliniek West, Den Haag | Bulder | R.M.A. | MSc. |
| Sint Anna Ziekenhuis | Cancrinus | E | MD |
| Maastricht Universitair Medisch Centrum | Clermonts | S.H.E.M. | MD, PhD |
| Amphia ziekenhuis | Coebergh - van den Braak | R.R.J. | MD, PhD |
| Meander Medisch Centrum | Consten | E.C.J. | MD, PhD |
| Maastricht Universitair Medisch Centrum | Coolsen | M.M.E. | MD, PhD |
| Amsterdam UMC | Daams | F | MD, PhD |
| Universitair Medisch Centrum Groningen (UMCG) | Dam, van | G.M. | MD, PhD |
| Zaans Medisch Centrum | Dekker | N.A.M. | MD, PhD |
| Ziekenhuisgroep Twente | Det, van | M.J. | MD, PhD |
| Amsterdam UMC | Dickhoff | C | MD, PhD |
| Bernhoven Ziekenhuis | Diepen, van | M.S. |  |
| Diakonessenhuis Utrecht | Dolmans | D | MD, PhD |
| Maasziekenhuis (Pantein) | Duijff | J.W. | MD |
| Universitair Medisch Centrum Groningen (UMCG) | Duis, ten | K | MD |
| Medisch Spectrum Twente | Duyn, van | E.B. | MD, MBA |
| Amsterdam UMC | Eshuis | W.J. | MD, PhD |
| Universitair Medisch Centrum Groningen (UMCG) | Etten, van | B | MD, PhD |
| Maasstadziekenhuis | Fioole | B | MD, PhD |
| Ziekenhuisgroep Twente | Folbert | E.C. | MANP |
| Medisch Spectrum Twente | Geelkerken | R.H. | MD, PhD |
| Maasziekenhuis (Pantein) | Gent, van | T | MD |
| Amsterdam UMC | Gisbertz | S.S. | MD, PhD |
| Medisch Spectrum Twente | Groot, de | R | MD |
| Erasmus Medisch Centrum | Grünhagen | D.J. | MD, PhD |
| Jeroen Bosch Ziekenhuis | Haelst, van | S.T.W. | MD, PhD |
| Thoraxcentrum Twente, Medisch Spectrum Twente | Halfwerk | F.R. | MD, PhD |
| Rode Kruis Ziekenhuis | Harlaar | N.J. | MD, PhD |
| Antoni van Leeuwenhoek Ziekenhuis - Nederlands Kanker Instituut (AvL-NKI) | Hartemink | K.J. | MD, PhD |
| Leids Universitair Medisch Centrum (LUMC) | Hartgrink | H.H. | MD, PhD |
| Universitair Medisch Centrum Utrecht | Hattum, van | E.H. | MD, PhD |
| Universitair Medisch Centrum Utrecht | Hazenberg | C.E.V.B. | MD, PhD |
| Elisabeth Tweesteden Ziekenhuis | Heisterkamp | J | MD, PhD |
| Admiraal De Ruyter Ziekenhuis | Helleman | J.N. | MD |
| Universitair Medisch Centrum Utrecht | Herwaarden, van | J.A. | MD, PhD |
| Universitair Medisch Centrum Utrecht | Hillegerberg, van | R | MD, PhD |
| Catharina Ziekenhuis | Himbeeck, van | M | MD |
| Catharina Ziekenhuis | Hingh, de | I.H.J.T. | MD, PhD |
| Martini Ziekenhuis | Hoed, van den | E | MD |
| Albert Schweitzer Ziekenhuis | Hoeven, van der | J.A.B. | MD, PhD |
| Reinier De Graaf Groep | Hoffmann | W.H. | MD |
| Amphia ziekenhuis | Huisman | S | MD |
| Elisabeth Tweesteden Ziekenhuis | Jansen | W.A. | MSc. |
| Catharina Ziekenhuis | Janssen | H.J.B. | MD |
| VieCuri Medisch Centrum | Janssen | R.J.L. | MD |
| VieCuri Medisch Centrum | Janssen-Maessen | S.H.P. | MSc. |
| Ziekenhuisgroep Twente | Jong, de | E | BSc. |
| Haaglanden Medisch Centrum | Jong, de | W.J. | MD |
| Meander Medisch Centrum | Jongen | J.M.J. | MD, PhD |
| Medisch Centrum Leeuwarden | Jonker | J | MD, PhD |
| Antoni van Leeuwenhoek Ziekenhuis - Nederlands Kanker Instituut (AvL-NKI) | Keessen | S.A. |  |
| Martini Ziekenhuis | Keizers | B | MD |
| Martini Ziekenhuis | Kelder | W | MD, PhD |
| Martini Ziekenhuis | Keller | B.P.J.A. | MD, PhD |
| Universitair Medisch Centrum St. Radboud | Klarenbeek | B | MD, PhD |
| Gelre Ziekenhuizen, Apeldoorn | Klemm | P.L. | MD |
| Ziekenhuisgroep Twente | Koenen | J.C. |  |
| Jeroen Bosch Ziekenhuis | Koeverden, van | I.D. | MD, PhD |
| Antoni van Leeuwenhoek Ziekenhuis - Nederlands Kanker Instituut (AvL-NKI) | Kok | N.F.M. | MD, PhD |
| Universitair Medisch Centrum St. Radboud | Kolk, van der | B.M. | MD, PhD |
| Admiraal De Ruyter Ziekenhuis | Koningswoud-Terhoeve | C.L. | MSc. |
| VieCuri Medisch Centrum | Kool | M | MD, PhD |
| Elisabeth Tweesteden Ziekenhuis | Kort- van Oudheusden, de | M | MSc. |
| Ziekenhuisgroep Twente | Kouwenhoven | E.A. | MD, PhD |
| Universitair Medisch Centrum Utrecht | Kroese | T.E. | MD |
| Antoni van Leeuwenhoek Ziekenhuis - Nederlands Kanker Instituut (AvL-NKI) | Kuhlmann | K.F.D. | MD, PhD |
| Universitair Medisch Centrum St. Radboud | Laarhoven, van | C.J.H.M. | MD, PhD |
| Erasmus Medisch Centrum | Lagarde | S.M. | MD, PhD |
| Sint Antonius Ziekenhuis, Nieuwegein | Lammeren, van | G.W. | MD, PhD |
| Elisabeth Tweesteden Ziekenhuis | Langenhoff | B.S. | MD, PhD |
| Rijnstate Ziekenhuis | Lardenoije | J.H.P. | MD, PhD |
| Universitair Medisch Centrum Groningen (UMCG) | Lases | S.S. | MD, PhD |
| Universitair Medisch Centrum Groningen (UMCG) | Lases | L | MD, PhD |
| Gelre Ziekenhuizen, Apeldoorn | Lastdrager | W | MD |
| Maxima Medisch Centrum | Leclercq | W.K.G. | MD, PhD |
| Maxima Medisch Centrum | Loos | M.J.A. | MD, PhD |
| Catharina Ziekenhuis | Luyer | M.D.P. | MD, PhD |
| Erasmus Medisch Centrum | Maat | A.P.W.M. | MD |
| Albert Schweitzer Ziekenhuis | Marres | G.M.H. | MD, PhD |
| Stichting Onze Lieve Vrouwe Gasthuis | Marsman | H.A. | MD, PhD |
| Ziekenhuisgroep Twente | Masselink | I |  |
| Universitair Medisch Centrum St. Radboud | Matthée | E | MD |
| Elisabeth Tweesteden Ziekenhuis | Matthijsen | R.M. | MD, PhD |
| Universitair Medisch Centrum Groningen (UMCG) | Meerdink | M | MD |
| Maastricht Universitair Medisch Centrum | Mees | B.M.E. | MD, PhD |
| Universitair Medisch Centrum Groningen (UMCG) | Meijer, de | V.E. | MD, PhD |
| Rode Kruis Ziekenhuis | Mekke | S | MD |
| Leids Universitair Medisch Centrum (LUMC) | Metselaar | A |  |
| Leids Universitair Medisch Centrum | Michiels | N | MD |
| Leids Universitair Medisch Centrum (LUMC) | Mieog | J.S.D. | MD, PhD |
| Medisch Spectrum Twente | Moekotte | N.L. | MSc. |
| Westfriesgasthuis / Dijklander ziekenhuis | Moes | D.E. | MD |
| Isala Klinieken | Molegraaf | M.J. | MD, PhD |
| Sint Antonius Ziekenhuis, Nieuwegein | Molenaar | I.Q. | MD, PhD |
| Slingeland Ziekenhuis | Mooy-Vermaat | S.P. |  |
| Franciscus Gasthuis & Vlietland | Morak | M | MD, PhD |
| Medisch Centrum Leeuwarden | Murrmann-Boonstra, | G | MD, PhD |
| Ziekenhuisgroep Twente | Navis | L.H. |  |
| Maasziekenhuis (Pantein) | Nes, de | L.C.F. | MD |
| Isala Klinieken | Nieuwenhuijs | V.B. | MD, PhD |
| Catharina Ziekenhuis | Nieuwenhuijzen | G.A.P. | MD, PhD |
| Canisius-Wilhelmina Ziekenhuis | Olofsen | N | MSc. |
| Haga Ziekenhuis | Ooms | S. | MD |
| Haaglanden Medisch Centrum | Oosterhuis, | J.W.A. | MD, PhD |
| Reinier De Graaf Groep | Orsini | M | MSc. |
| Gelre Ziekenhuizen, Apeldoorn | Palamba | H.W. |  |
| Haga Ziekenhuis | Pasveer | E.H. | MD |
| Isala Klinieken | Patijn | G.A. | MD, PhD |
| Nij Smellinghe | Pereboom | I.T.A. | MD, PhD |
| Universitair Medisch Centrum St. Radboud | Peters | N | Master of advanced nursing |
| Universitair Medisch Centrum Utrecht | Petri | B.J. | MD, PhD |
| Medisch Centrum Leeuwarden | Pierie | J.P.E.N. | MD, PhD |
| Gelderse Vallei | Ponfoort | E.D. | MD |
| Universitair Medisch Centrum Groningen (UMCG) | Pranger | B | MD |
| Martini Ziekenhuis | Pultrum | B.B. | MD, PhD |
| Catharina Ziekenhuis | Ramaekers | M | MD |
| Stichting Onze Lieve Vrouwe Gasthuis | Rassam | F | MD, PhD |
| Rijnstate Ziekenhuis | Reijnen | M.M.P.J. | MD, PhD |
| Amphia ziekenhuis | Rijken | A.M. | MD, PhD |
| Universitair Medisch Centrum St. Radboud | Rosman | C | MD, PhD |
| Martini Ziekenhuis | Rossum, van | E |  |
| Jeroen Bosch Ziekenhuis | Rots | M.L. | MD, PhD |
| Universitair Medisch Centrum Groningen (UMCG) | Ruiter | S | MSc. |
| Universitair Medisch Centrum Groningen (UMCG) | Rurenga | M | MD |
| Antoni van Leeuwenhoek Ziekenhuis - Nederlands Kanker Instituut (AvL-NKI) | Sandick, van | J.W. | MD, PhD |
| Sint Antonius Ziekenhuis, Nieuwegein | Santvoort, van | H.C. | MD, PhD |
| Rijnstate Ziekenhuis | Schaik, van | P | MD, PhD |
| Slingeland Ziekenhuis | Scharn | D.M. | MD, PhD |
| Leids Universitair Medisch Centrum (LUMC) | Schipper | I.B. | MD |
| Bernhoven Ziekenhuis | Schoonderwoerd | L | MD |
| Sint Jansdal Ziekenhuis | Schouten- van der Velden. | A.P. | MD, PhD |
| Universitair Medisch Centrum St. Radboud | Schroen | A.M.A. | MD, PhD |
| Maastricht Universitair Medisch Centrum | Schurink | G.W.H | MD, PhD |
| Maxima Medisch Centrum | Sijmons | J.M.L. | MD |
| Erasmus Medisch Centrum | Sluis, van der | P.C. | MD, PhD |
| Rijnstate Ziekenhuis | Smeets | L | MD, PhD |
| Westfriesgasthuis / Dijklander ziekenhuis | Smit | J.W. | MD |
| Zuyderland Medisch centrum | Sosef | M.N. | MD, PhD |
| Catharina Ziekenhuis | Stacie | R | MD |
| Medisch Spectrum Twente | Stam | A | MD |
| Rijnstate Ziekenhuis | Sterkenburg, van | S.M.M. | MD |
| Leids Universitair Medisch Centrum (LUMC) | Steur, de | W.O. | MD |
| Bernhoven Ziekenhuis | Stevens | C.T. | MD |
| Universitair Medisch Centrum St. Radboud | Stommel | M.W.J. | MD, PhD |
| Zuyderland Medisch centrum | Stoot | J.H.M.B. | MD, PhD |
| Bravis Ziekenhuis | Susa | D | MD, PhD |
| Nederlandse Obesitas Kliniek West, Den Haag | Swank | D.J. | MD, PhD |
| Medisch Centrum Leeuwarden | Swart | J |  |
| Gelderse Vallei | Sybrandy | J.E.M. | MD |
| Deventer Ziekenhuis | Talsma | A.K. | MD |
| Maasstadziekenhuis | Terlouw | E.C. |  |
| Deventer Ziekenhuis | Tissink | M.W. | MD |
| Maasziekenhuis (Pantein) | Toonen | D |  |
| Universitair Medisch Centrum Utrecht | Toorop | R.J. | MD, PhD |
| Albert Schweitzer Ziekenhuis | Tozzi | F | MD |
| Groene Hart Ziekenhuis | Tseng | L.N.L. | MD |
| Haaglanden Medisch Centrum | Tummers | Q | MD, PhD |
| Maxima Medisch Centrum | Vaes | R.H.D. | MD, PhD |
| Maxima Medisch Centrum | Vancoillie | P.J. | MD |
| Antoni van Leeuwenhoek Ziekenhuis - Nederlands Kanker Instituut (AvL-NKI) | Veenhof | A.A. | MD, PhD |
| Haga Ziekenhuis | Veger | H.T.C. | MD |
| Isala Klinieken | Veldt, van der | M.E.V. |  |
| VieCuri Medisch Centrum | Verberght | H | MD |
| Rode Kruis Ziekenhuis | Verhaakt | T | MD |
| Universitair Medisch Centrum St. Radboud | Verhagen | A.F.T.M. | MD, PhD |
| Meander Medisch Centrum | Verheijen | P.M. | MD, PhD |
| Wilhelmina Ziekenhuis | Vierhout | B.P. | MD, PhD |
| Universitair Medisch Centrum St. Radboud | Vijver, van der | R | MD, PhD |
| Albert Schweitzer Ziekenhuis | von Meyenfeldt | E.M. | MD |
| Sint Antonius Ziekenhuis, Nieuwegein | Vos | A.W.F. | MD, PhD |
| Wilhelmina Ziekenhuis | Vos, de | B. | MD |
| Medisch Spectrum Twente | Voshaar | A | MANP |
| Erasmus Medisch Centrum | Waalboer | R.B. | MSc. |
| Antoni van Leeuwenhoek Ziekenhuis - Nederlands Kanker Instituut (AvL-NKI) | Wals | A | MD |
| Universitair Medisch Centrum St. Radboud | Warlé | M.C. | MD, PhD |
| Meander Medisch Centrum | Weel, van | V | MD, PhD |
| Westfriesgasthuis / Dijklander ziekenhuis | Wiersema | A.M. | MD, PhD |
| Universitair Medisch Centrum Groningen (UMCG) | Wiersma | A | MD |
| Martini Ziekenhuis | Wijma | A | MD |
| Universitair Medisch Centrum Groningen (UMCG) | Wijsmuller | A.R. | MD, PhD |
| Haaglanden Medisch Centrum | Wilden, van der | G.M. | MD, PhD |
| Universitair Medisch Centrum St. Radboud | Wilt, de | J.H.W. | MD, PhD |
| Sint Jansdal Ziekenhuis | Woensdregt | K | MD |
| Amsterdam UMC, | Zandbergen | H.R. | MD, PhD, MBA |
| Dutch COVIDSurg Collaborative Studygroup | | | |

**Appendix S2: Supplementary Tables**

**Table S1a**: An overview of variables registered in selected clinical audits only.

|  | **DATO** | **DCRA** | **DHBA** | **DHFA** | **DLCA** | **DSAA** | **DPCA** | **DUCA** | |
| --- | --- | --- | --- | --- | --- | --- | --- | --- | --- |
| BMI | x | x | x |  | x |  | x | x | |
| Charlson comorbidity scores | x | x | x |  | x |  | x | x | |
| ASA grade | x | x | x |  | x |  | x | x | |
| Timing of surgery (acute/planned) | x | x |  | x | x | x | x | x | |
| ICU stay | x | x | x |  | x | x | x | x | |
| ICU length of stay | x | x | x |  | x | x |  | x | |
| Readmission | x | x | x |  | x | x | x | x | |
| In hospital or 30-day mortality | x |  | x | x | x | x | x | x | |
| In hospital or 90-day mortality |  | x |  |  |  |  |  |  | |
| Need for reintervention | x | x | x |  | x | x | x | x | |
| Complications 30-day | x |  | x |  | x |  | x | x | |
| Complications 90-days |  | x |  |  |  |  |  |  | |
| Overview of variables registered in selected clinical audits only are indicated by x.  Abbreviations: DATO = Dutch Audit for Treatment of Obesity; DCRA = Dutch Colorectal Cancer Audit; DHBA = Dutch Hepato Biliary Audit; DHFA = Dutch Hip Fracture Audit; DLCA = Dutch Lung Cancer Audit; DSAA = Dutch Surgical Aneurysm Audit; DPCA = Dutch Pancreatic Cancer audit; DUCA = Dutch Upper Gastrointestinal Cancer Audit; BMI = Body Mass Index; ASA Score = American Society of Anesthesiologists Grade; ICU = Intensive Care Unit; Readmission within 30-days after discharge. | | | | | | | | |  |

| **registry** | DLCA | |  |  | DUCA | | DPCA | |  | DHBA | |  |  | DCRA | | DHFA | DSAA | |  |  | DATO | |  |
| --- | --- | --- | --- | --- | --- | --- | --- | --- | --- | --- | --- | --- | --- | --- | --- | --- | --- | --- | --- | --- | --- | --- | --- |
| **Specific procedure** | Resection lungcarcinoma | Mediastinoscopy | Metastatectomy | Other lung surgery | Gastric procedure | Oesphagic procedure | PPPD/Whipple | Pancreas tail resection | Other pancreatic surgery | Colorectal liver metastases | Hepatocellular-carcinoma | Cholangio-carcinoma | Benigne liver tumour | Colon procedure | Anorectal procedure | Hip fractures | Abdominal Aneurysm - EVAR | Abdominal Aneurysm - open | Thoracic Aneurysm - TEVAR | Thoracic Aneurysm - open | Roux and Y gastric bypass | Gastric Sleeve | Other bariatric surgery |
| BMI |  | NR | NR | NR |  |  |  |  |  |  |  |  |  |  |  | NR | NR | NR | NR | NR |  |  |  |
| comorbdities | NR* | NR | NR^ | NR^ |  |  |  |  |  |  |  |  |  |  |  | NR | NR* | NR* | NR* | NR* |  |  |  |
| Charlson comorbidity scores |  |  |  |  |  |  |  |  |  |  |  |  |  |  |  | NR | NR | NR | NR | NR |  |  |  |
| ASA grade |  |  |  |  |  |  |  |  |  |  |  |  |  |  |  |  | NR | NR | NR | NR |  |  |  |
| Readmission* |  |  |  |  |  |  |  |  |  | NR * | NR * | NR * | NR * |  |  |  |  |  |  |  |  |  |  |
| Severe complications ^#^ |  |  |  |  |  |  |  |  |  |  |  |  |  |  |  | NR | NR | NR | NR | NR |  |  |  |
| ICU stay |  |  |  |  |  |  |  |  |  |  |  |  |  |  |  | NR |  |  |  |  |  |  |  |
| Clinical TNM stage |  |  |  |  |  |  |  |  |  | NR | NR | NR | NR |  |  |  |  |  |  |  |  |  |  |
| Time to surgery |  | NR | NR | NR |  |  |  |  |  |  |  |  |  |  |  |  |  |  |  |  |  |  |  |
| Overview of variables registered in selected clinical audits per specific type of procedure are indicated by NR (Not registered). NR* = not registered in 2018. NR ^= A selection of comorbidities was registered in 2018. # Not all variables to calculate severe complications defined as need for reintervention, ICU-admission and/or death were available. Abbreviations: DATO = Dutch Audit for Treatment of Obesity; DCRA = Dutch Colorectal Cancer Audit; DHBA = Dutch Hepato Biliary Audit; DHFA = Dutch Hip Fracture Audit; DLCA = Dutch Lung Cancer Audit; DSAA = Dutch Surgical Aneurysm Audit; DPCA = Dutch Pancreatic Cancer audit; DUCA = Dutch Upper Gastrointestinal Cancer Audit; BMI = Body Mass Index; ASA Score = American Society of Anesthesiologists Grade; ICU = Intensive Care Unit; Readmission within 30-days after discharge; Complications scored by Clavien Dindo. BMI = Body Mass Index; ASA Score = American Society of Anesthesiologists Grade; ICU = Intensive Care Unit | | | | | | | | | | | | | | | | | | | | | | | |

**Table S1b**: An overview of variables registered per specific procedure. Non-registered variables were not included in analysis.

**Tables S2a–S2h**

**Table S2a:** An overview of patients’ characteristics of patients registered in the Surgical Dutch Lung Cancer Audit - (DLCA-S) undergoing surgery between 1^st^ January 2018 and the 31^st^ of December 2020.

| **Characteristics DLCA-S** | **2018**  n=2 340 | **2019**  n=2 444 | **2020**  n=2 133 | **p-value** |
| --- | --- | --- | --- | --- |
| Age median [IQR] | 65.00 [55.00 71.00] | 65.00 [54.00 72.00] | 64.00 [53.00 72.00] | 0.70 |
| Age n(%) |  |  |  | 0.15 |
| <50 | 422 (18.0) | 489 (20.0) | 415 (19.5) |  |
| 50-64 | 733 (31.3) | 717 (29.3) | 688 (32.3) |  |
| 65-79 | 1077 (46.0) | 1134 (46.4) | 926 (43.4) |  |
| >80 | 108 (4.6) | 104 (4.3) | 104 (4.9) |  |
|  |  |  |  |  |
| Sex n (%) |  |  |  | 0.42 |
| Male | 1 406 (60.1) | 1 424 (58.3) | 1 253 (58.7) |  |
| Female | 934 (39.9) | 1020 (41.7) | 880 (41.3) |  |
|  |  |  |  |  |
| BMI n(%) |  |  |  | 0.09 |
| BMI 0-19 | 43 (1.8) | 50 (2.0) | 59 (2.8) |  |
| BMI 20-25 | 309 (13.2) | 287 (11.7) | 242 (11.3) |  |
| BMI 25-30 | 254 (10.9) | 282 (11.5) | 267 (12.5) |  |
| BMI >30 | 142 (6.1) | 125 (5.1) | 118 (5.5) |  |
| Not-registered* | 1 359 (59,1) | 1489 (60.9) | 1 264 (59.2) |  |
| Missing | 232(9.9) | 210 (8.5) | 183 (8.6) |  |
|  |  |  |  |  |
| Comorbidities present n(%) | 728 (31.1) | 1 384 (56.6) | 1 150 (53.9) | **<0.001** |
| Not registered* | 1357 (58.0) | - | - |  |
| Missing |  | 73 (3.0) | 62 (2.9) |  |
|  |  |  |  |  |
| Charlson Comorbidity Index (CCI) |  |  |  | **<0.001** |
| 0-1 | 2 042 (87.3) | 1 081 (44.2) | 999 (46.8) |  |
| 2+ | 298 (12.7) | 1 363 (55.8) | 1 134 (53.2) |  |
|  |  |  |  |  |
| ASA grade n(%) |  |  |  | 0.19 |
| ASA grade 1-2 | 1 320 (56.4) | 1 396 (57.1) | 1 162 (54.5) |  |
| ASA grade 3-5 | 988 (42.2) | 1 003 (41.0) | 941 (44.1) |  |
| Missing | 32 (1.4) | 45 (1.8) | 30 (1.4) |  |
|  |  |  |  |  |
| Indication for surgery n(%) |  |  |  | 0.08 |
| Oncologic | 1 159 (49.5) | 1 152 (47.1) | 1 072 (50.3) |  |
| Non-oncologic | 1 181 (50.5) | 1 292 (52.9) | 1 061 (49.7) |  |
|  |  |  |  |  |
| Perioperative SARS-Cov-2 infection n(%) |  |  | 13 (0.56) |  |
| Preoperative confirmed | - | - | 7 (0.3) |  |
| Postoperative confirmed | - | - | 9 (0.4) |  |
|  |  |  |  |  |
| Audit specific procedure n(%) |  |  |  | **0.007** |
| Resection lungcarcinoma | 981 (41.9) | 955 (39.1) | 869 (40.7) |  |
| Mediastinoscopy | 418 (17.9) | 407 (16.7) | 387 (18.1) |  |
| Metastatectomy | 178 (7.6) | 197 (8.1) | 203 (9.5) |  |
| Other lung surgery | 763 (32.6) | 885 (36.2) | 1. 1.6) |  |
| * Not registered means that this variable is not registered for mediastinoscopy, metastatectomy and other lung surgery.  Abbreviations: BMI = Body Mass Index; ASA Score = American Society of Anesthesiologists Grade; | | | | |

**Table S2b:** An overview of patients’ characteristics of patients registered in the Dutch Upper Gastrointestinal Cancer Audit (DUCA) undergoing surgery between 1^st^ January 2018 and the 31^st^ of December 2020.

| **Characteristics DUCA** | **2018**  n=830 | **2019**  n=811 | **2020**  n=931 | **p-value** |
| --- | --- | --- | --- | --- |
| Age median [IQR] | 69.00 [62.00 75.00] | 68.00 [62.00 74.00] | 69.00 [62.00 74.50] | 0.57 |
| Age n(%) |  |  |  | 0.48 |
| <50 | 36 (4.3) | 34 (4.2) | 57 (6.1) |  |
| 50-64 | 236 (28.4) | 237 (29.2) | 257 (27.6) |  |
| 65-79 | 484 (58.3) | 471 (58.1) | 527 (56.6) |  |
| >80 | 74 (8.9) | 69 (8.5) | 90 (9.7) |  |
|  |  |  |  |  |
| Sex n(%) |  |  |  | 0.53 |
| Male | 616 (74.2) | 594 (73.2) | 669 (71.9) |  |
| Female | 214 (25.8) | 217 (26.8) | 262 (28.1) |  |
| Missing | 0 | 0 | 0 |  |
|  |  |  |  |  |
| BMI n(%) |  |  |  | 0.26 |
| BMI 0-19 | 57 (6.9) | 52 (6.4) | 65 (7.0) |  |
| BMI 20-25 | 327 (39.4) | 317 (39.1) | 388 (41.7) |  |
| BMI 25-30 | 313 (37.7) | 293 (36.1) | 347 (37.3) |  |
| BMI >30 | 129 (15.5) | 138 (17.0) | 126 (13.5) |  |
| Missing | 4 (0.5) | 11 (1.4) | 5 (0.5) |  |
|  |  |  |  |  |
| Comorbidities present n(%) | 442 (53.3) | 432 (53.3) | 536 (57.6) | **<0.001** |
|  |  |  |  |  |
| Charlson Comorbidity Index (CCI) |  |  |  | 0.13 |
| 0-1 | 583 (70.2) | 556 (68.6) | 613 (65.8) |  |
| 2+ | 247 (29.8) | 255 (31.4) | 318 (34.2) |  |
|  |  |  |  |  |
| ASA grade n(%) |  |  |  | **0.01** |
| ASA grade 1-2 | 577 (69.5) | 514 (63.5) | 582 (63.5) |  |
| ASA grade 3-5 | 253 (30.5) | 296 (36.5) | 335 (36.5) |  |
|  |  |  |  |  |
| Urgency of surgery n(%) |  |  |  | 0.17 |
| Planned | 817 (98.4) | 804 (99.1) | 910 (97.7) |  |
| Acute | 13 (1.6) | 7 (0.9) | 20 (2.1) |  |
|  |  |  |  |  |
| Perioperative SARS-Cov-2 infection n(%) |  |  | 17 (1.8) |  |
| Preoperative confirmed | - | - | 4 (1.4) |  |
| Postoperative confirmed | - | - | 14 (4.6) |  |
|  |  |  |  |  |
| Audit specific procedure n(%) |  |  |  | 0.24 |
| Gastric procedure | 276 (33.3) | 261 (32.2) | 334 (35.9) |  |
| Oesophageal procedure | 553 (66.7) | 549 (67.8) | 596 (64.1) |  |
| Abbreviations: BMI = Body Mass Index; ASA Score = American Society of Anesthesiologists Grade; | | | | |

**Table S2c**: An overview of patients’ characteristics of patients registered in the Dutch Pancreatic Cancer Audit (DPCA) undergoing surgery between 1^st^ January 2018 and the 31^st^ of December 2020.

| **Characteristics DPCA** | **2018**  n=626 | **2019**  n=636 | **2020**  n=663 | **p-value** |
| --- | --- | --- | --- | --- |
| Age median [IQR] | 67.00 [59.00 74.00] | 68.00 [61.00 74.00] | 68.00 [59.00 75.00] | 0.06 |
| Age n(%) |  |  |  | **0.04** |
| <50 | 66 (10.5) | 53 (8.3) | 43 (6.5) |  |
| 50-64 | 187 (29.9) | 180 (28.3) | 201 (30.3) |  |
| 65-79 | 332 (53.0) | 357 (56.1) | 353 (53.2) |  |
| >80 | 41 (6.5) | 46 (7.2) | 66 (10.0) |  |
|  |  |  |  |  |
| Sex n(%) |  |  |  | 0.06 |
| Male | 301 (48.1) | 344 (54.1) | 353 (53.2) |  |
| Female | 325 (51.9) | 292 (45.9) | 310 (46.8) |  |
|  |  |  |  |  |
| BMI n(%) |  |  |  | 0.17 |
| BMI 0-19 | 57 (9.1) | 45 (7.1) | 40 (6.0) |  |
| BMI 20-25 | 266 (42.5) | 286 (45.0) | 289 (43.6) |  |
| BMI 25-30 | 215 (34.3) | 203 (31.9) | 220 (33.2) |  |
| BMI >30 | 80 (12.8) | 90 (14.2) | 109 (16.4) |  |
| Missing | 8 (1.3) | 12 (1.9) | 5 (0.8) |  |
|  |  |  |  |  |
| Comorbidities present n(%) | 331 (54.4) | 395 (63.5) | 420 (65.1) | <**0.001** |
| Missing | 0 (0.0) | 0 (0.0) | 0 (0.0) |  |
|  |  |  |  |  |
| Charlson Comorbidity Index (CCI) |  |  |  | **<0.001** |
| 0-1 | 437 (71.8) | 386 (62.1) | 392 (60.8) |  |
| 2+ | 172 (28.2) | 236 (37.9) | 253 (39.2) |  |
| Missing | 0 | 0 | 0 |  |
|  |  |  |  |  |
| ASA grade n(%) |  |  |  | **<0.001** |
| ASA grade 1-2 | 489 (78.1) | 417 (65.6) | 413 (62.3) |  |
| ASA grade 3-5 | 136 (21.7) | 212 (33.3) | 231 (34.8) |  |
| Missing | 1 (0.2) | 7 (1.1) | 19 (2.9) |  |
|  |  |  |  |  |
| Indication for surgery n(%) |  |  |  | 0.27 |
| Oncologic | 605 (96.6) | 614 (96.5) | 649 (97.9) |  |
| Non-oncologic | 21 (3.4) | 22 (3.5) | 14 (2.1) |  |
|  |  |  |  |  |
| Perioperative SARS-Cov-2 infection n(%) |  |  | 9 (0.7) |  |
| Preoperative confirmed | - | - | 2 (0.3) |  |
| Postoperative confirmed | - | - | 8 (0.6) |  |
|  |  |  |  |  |
| Audit specific procedure n(%) |  |  |  | 0.08 |
| PPPD/Whipple | 420 (67.1) | 432 (67.9) | 476 (71.8) |  |
| Pancreas tail resection | 111 (17.7) | 113 (17.8) | 120 (18.1) |  |
| Other | 95 (15.2) | 91 (14.3) | 67 (10.1) |  |
| Abbreviations: BMI = Body Mass Index; ASA Score = American Society of Anesthesiologists Grade; PPPD: pylorus-preserving pancreaticoduodenectomy. | | | | |

**Table S2d:** An overview of patient’s characteristics of patients registered in the Dutch Hepato Biliary Audit (DHBA) undergoing surgery between 1^st^ January 2018 and the 31^st^ of December 2020.

| **Characteristics DHBA** | **2018**  n=1 190 | **2019**  n=1 325 | **2020**  n=1 215 | **p-value** |
| --- | --- | --- | --- | --- |
| Age median [IQR] | 65.00 [56.00 73.00] | 66.00 [58.00 73.00] | 66.00 [57.00 73.50] | **0.01** |
| Age n(%) |  |  |  | **0.04** |
| <50 | 162 (13.6) | 138 (10.4) | 154 (12.7) |  |
| 50-64 | 422 (35.5) | 444 (33.5) | 424 (34.9) |  |
| 65-79 | 541 (45.5) | 641 (48.4) | 551 (45.3) |  |
| >80 | 65 (5.5) | 102 (7.7) | 86 (7.1) |  |
|  |  |  |  |  |
| Sex n(%) |  |  |  | 0.629 |
| Male | 700 (58.8) | 810 (61.1) | 721 (59.3) |  |
| Female | 489 (41.1) | 515 (38.9) | 493 (40.6) |  |
| Missing | 1(0.1) | 0 | 0 |  |
|  |  |  |  |  |
| BMI n(%) |  |  |  | 0.419 |
| BMI 0-19 | 53 (4.5) | 64 (4.8) | 59 (4.9) |  |
| BMI 20-25 | 428 (36.0) | 446 (33.7) | 402 (33.1) |  |
| BMI 25-30 | 477 (40.1) | 521 (39.3) | 492 (40.5) |  |
| BMI >30 | 226 (19.0) | 286 (21.6) | 248 (20.4) |  |
| Missing | 6 (0.5) | 8 (0.6) | 14 (1.2) |  |
|  |  |  |  |  |
| Comorbidities present n(%) | 576 (48.4) | 922 (69.6) | 748 (61.6) | **<0.001** |
| Missing | 9 (0.8) | 40 (3.0) | 25 (2.1) |  |
|  |  |  |  |  |
| Charlson Comorbidity Index (CCI) |  |  |  | **<0.001** |
| 0-1 | 853 (71.7) | 542 (40.9) | 637 (52.4) |  |
| 2+ | 337 (28.3) | 783 (59.1) | 578 (47.6) |  |
|  |  |  |  |  |
| ASA grade n(%) |  |  |  | **<0.001** |
| ASA grade 1-2 | 851 (71.5) | 815 (61.5) | 792 (65.2) |  |
| ASA grade 3-5 | 337 (28.3) | 510 (38.5) | 422 (34.7) |  |
| Missing | 2 (0.2) | 0 (0.0) | 1 (0.1) |  |
|  |  |  |  |  |
| Indication for surgery n(%) |  |  |  | 0.27 |
| Oncological | 1 094 (91.9) | 1 240 (93.6) | 1 127 (92.8) |  |
| Non-oncological | 96 (8.1) | 85 (6.4) | 88 (7.2) |  |
|  |  |  |  |  |
| Perioperative SARS-Cov-2 infection n(%) |  |  | 3 (0.25) |  |
| Preoperative confirmed | - | - | 0 (0.0) |  |
| Postoperative confirmed | - | - | 3 (0.25) |  |
|  |  |  |  |  |
| Audit specific indication for surgery n(%) |  |  |  | **<0.001** |
| Colorectal liver metastases | 889 (74.7) | 884 (66.7) | 854 (70.3) |  |
| Hepatocellular-carcinoma | 116 (9.7) | 272 (20.5) | 189 (15.6) |  |
| Cholangio-carcinoma | 89 (7.5) | 84 (6.3) | 84 (6.9) |  |
| Benigne liver tumour | 96 (8.1) | 85 (6.4) | 88 (7.2) |  |
| Abbreviations: BMI = Body Mass Index; ASA Score = American Society of Anesthesiologists Grade; | | | | |

**Table S2e**: An overview of patient’s characteristics of patients registered in the Dutch Colo Rectal Audit (DCRA) undergoing surgery between 1^st^ January 2018 and the 31^st^ of December 2020.

| **Characteristics DCRA** | **2018**  n=2 572 | **2019**  n=2 355 | **2020**  n=1 933 | **p-value** |
| --- | --- | --- | --- | --- |
| Age median [IQR] | 72.00 [62.00 76.00] | 70.00 [61.00 77.00] | 72.00 [62.00 78.00] | **0.03** |
| Age n(%) |  |  |  | **0.01** |
| <50 | 96 (3.7) | 105 (4.5) | 81 (4.2) |  |
| 50-64 | 714 (27.8) | 652 (27.7) | 504 (26.1) |  |
| 65-79 | 1317 (51.2) | 1147 (48.7) | 932 (48.2) |  |
| >80 | 444 (17.3) | 451 (19.2) | 416 (21.5) |  |
|  |  |  |  |  |
| Sex n(%) |  |  |  | **0.03** |
| Male | 1 469 (57.1) | 1 257 (53.4) | 1 062 (54.9) |  |
| Female | 1 102 (42.9) | 1 098 (46.6) | 871 (45.1) |  |
|  |  |  |  |  |
| BMI n(%) |  |  |  | **<0.001** |
| BMI 0-19 | 82 (3.2) | 96 (4.1) | 110 (5.7) |  |
| BMI 20-25 | 818 (31.8) | 830 (35.2) | 708 (36.6) |  |
| BMI 25-30 | 1 050 (40.8) | 973 (41.3) | 749 (38.7) |  |
| BMI >30 | 517 (20.1) | 420 (17.8) | 350 (18.1) |  |
| Missing | 104 (4.0) | 36 (1.5) | 16 (0.8) |  |
|  |  |  |  |  |
| Comorbidities present n(%) | 1 109 (43.1) | 988 (42.0) | 869 (45.0) | **<0.001** |
|  |  |  |  |  |
| Charlson Comorbidity Index (CCI) |  |  |  | 0.11 |
| 0-1 | 1 466 (57.0) | 1 371 (58.2) | 1 064 (55.0) |  |
| 2+ | 1 105 (43.0) | 984 (41.8) | 869 (45.0) |  |
|  |  |  |  |  |
| ASA grade n(%) |  |  |  | **0.001** |
| ASA grade 1-2 | 1 821 (70.8) | 1 667 (70.8) | 1 281 (66.3) |  |
| ASA grade 3-5 | 750 (29.2) | 688 (29.2) | 652 (33.7) |  |
| Missing | 1 (0.0) | 0 (0.0) | 0 (0.0) |  |
|  |  |  |  |  |
| Indication for surgery n(%) |  |  |  | **<0.001** |
| Oncologic | 2 572 (100) | 2 355 (100) | 1 933 (100) |  |
| Non-oncologic | 0 | 0 | 0 |  |
|  |  |  |  |  |
| Urgency of surgery n(%) |  |  |  | **0.001** |
| Acute | 165 (6.4) | 149 (6.3) | 166 (8.6) |  |
| Planned | 2 406 (93.6) | 2 201 (93.5) | 1 767 (91.4) |  |
|  |  |  |  |  |
| Perioperative SARS-Cov-2 infection n(%) |  |  | 8 (0.41) |  |
| Preoperative confirmed | - | - | 2 (0.10) |  |
| Postoperative confirmed | - | - | 7 (0.35) |  |
|  |  |  |  |  |
| Audit specific procedure n(%) |  |  |  | **0.03** |
| Colon procedure | 1 863 (74.9) | 1 792 (78.1) | 1 450 (77.3) |  |
| Anorectal procedure | 623 (25.1) | 502 (21.9) | 426 (22.7) |  |
| Abbreviations: BMI = Body Mass Index; ASA Score = American Society of Anesthesiologists Grade; | | | | |

**Table S2f:** An overview of patient’s characteristics of patients registered in the Dutch Hip Fracture Audit (DHFA) undergoing surgery between 1^st^ January 2018 and the 31^st^ of December 2020.

| **Characteristics DHFA** | **2018**  n=1 994 | **2019**  n=2 226 | **2020**  n=2 138 | **p-value** |
| --- | --- | --- | --- | --- |
| Age median [IQR] | 81.00 [72.00 87.00] | 81.00 [72.00 87.00] | 81.00 [72.00 87.00] | 0.83 |
| Age n(%) |  |  |  | 0.07 |
| <50 | 46 (2.3) | 69 (3.1) | 61 (2.9) |  |
| 50-64 | 185 (9.3) | 261 (11.7) | 213 (10.0) |  |
| 65-79 | 651 (32.6) | 668 (30.0) | 668 (31.2) |  |
| >80 | 1 112 (55.8) | 1 228 (55.2) | 1 196 (55.9) |  |
|  |  |  |  |  |
| Sex n(%) |  |  |  | 0.26 |
| Male | 696 (34.9) | 749 (33.6) | 732 (34.2) |  |
| Female | 1 296 (65.0) | 1 475 (66.3) | 1 399 (65.4) |  |
|  |  |  |  |  |
| ASA grade n(%) |  |  |  | **<0.001** |
| ASA grade 1-2 | 781 (39.2) | 883 (39.7) | 798 (37.3) |  |
| ASA grade 3-5 | 1 043 (52.3) | 1 336 (60.0) | 1 333 (62.3) |  |
| Missing | 170 (8.5) | 7 (0.3) | 7 (0.3) |  |
|  |  |  |  |  |
| Urgency of Surgery n(%) |  |  |  | 1 |
| Planned | 0 (0) | 0 (0) | 0 (0) |  |
| Acute | 1 994 (100.0) | 2 226 (100.0) | 2 138 (100.0) |  |
|  |  |  |  |  |
| Perioperative SARS-Cov-2 infection n(%) |  |  | 17 (1.03) |  |
| Preoperative confirmed | - | - | 10 (0.5) |  |
| Postoperative confirmed | - | - | 14 (0.7) |  |
| Abbreviations: BMI = Body Mass Index; | | | | |

**Table S2g**: An overview of patient’s characteristics of patients registered in the Dutch Surgical Aneurysm Audit (DSAA) undergoing surgery between 1^st^ January 2018 and the 31^st^ of December 2020.

| **Characteristics DSAA** | **2018**  n=1 772 | **2019**  n=1 761 | **2020**  n=1 595 | **p-value** |
| --- | --- | --- | --- | --- |
|  |  |  |  |  |
| Age median [IQR] | 74.00 [68.00 79.00] | 74.00 [69.00 80.00] | 74.00 [68.00 79.00] | 0.47 |
| Age n(%) |  |  |  | 0.40 |
| <50 | 19 (1.1) | 27 (1.5) | 21 (1.3) |  |
| 50-64 | 236 (13.3) | 209 (11.9) | 224 (14.0) |  |
| 65-79 | 1 095 (61.8) | 1 074 (61.0) | 961 (60.3) |  |
| >80 | 422 (23.8) | 451 (25.6) | 389 (24.4) |  |
|  |  |  |  |  |
| Sex n(%) |  |  |  | **0.01** |
| Male | 1510 (85.2) | 1442 (81.9) | 1342 (84.1) |  |
| Female | 262 (14.8) | 319 (18.1) | 251 (15.7) |  |
| Missing | 0 (0.0) | 0 (0.0) | 2 (0.1) |  |
|  |  |  |  |  |
| Comorbidities present n(%) | 10 (0.6) | 1 428 (81.1) | 1 289 (80.8) | **<0.001** |
| not registered* | 1 716 (96.8) | - | - |  |
| Missing | 0 | 0 (0) | 0 (0) |  |
|  |  |  |  |  |
| Urgency of Surgery n(%) |  |  |  | 0.52 |
| Acute | 427 (24.1) | 405 (23.0) | 388 (24.3) |  |
| Planned | 1 345 (75.9) | 1 356 (77.0) | 1 206 (75.6) |  |
|  |  |  |  |  |
| Perioperative SARS-Cov-2 infection n(%) |  |  | 8 (0.5) |  |
| Preoperative confirmed | - | - | 2 (0.1) |  |
| Postoperative confirmed | - | - | 6 (0.4) |  |
|  |  |  |  |  |
| Audit specific procedure n(%) |  |  |  | 0.83 |
| Abdominal Aneurysm - EVAR | 1 087 (61.3) | 1 070 (60.8) | 1 004 (62.9) |  |
| Abdominal Aneurysm - open | 469 (26.5) | 481 (27.3) | 424 (26.6) |  |
| Thoracic Aneurysm - TEVAR | 122 (6.9) | 118 (6.7) | 87 (5.5) |  |
| Thoracic Aneurysm - open | 69 (3.9) | 68 (3.9) | 1. 3.6) |  |

*Not registered means that this variable is not registered in 2018 for patients registered in the DSAA

**Table S2h**: An overview of patient’s characteristics of patients registered in the Dutch Audit for Treatment of Obesity (DATO) undergoing surgery between 1^st^ January 2018 and the 31^st^ of December 2020.

| **Characteristics DATO** | **2018**  n=2 661 | **2019**  n=2 599 | **2020**  n=1 543 | **p-value** |
| --- | --- | --- | --- | --- |
| Age median [IQR] | 47.00 [36.00 54.00] | 47.00 [36.00 54.00] | 46.00 [35.00 54.00] | **0.05** |
| Age n(%) |  |  |  | 0.15 |
| <50 | 1 620 (60.9) | 1 533 (59.0) | 963 (62.4) |  |
| 50-64 | 986 (37.1) | 996 (38.3) | 542 (35.1) |  |
| 65-79 | 55 (2.1) | 70 (2.7) | 38 (2.5) |  |
| >80 | 0 (0.0) | 0 (0.0) | 0 (0.0) |  |
|  |  |  |  |  |
| Sex n(%) |  |  |  | 0.06 |
| Male | 549 (20.6) | 583 (22.4) | 363 (23.7) |  |
| Female | 2 111 (79.4) | 2 016 (77.6) | 1 168 (76.3) |  |
|  |  |  |  |  |
| BMI n(%) |  |  |  | **0.005** |
| BMI 0-19 | 0 (0.0) | 0 (0.0) | 0 (0.0) |  |
| BMI 20-25 | 3 (0.1) | 4 (0.2) | 3 (0.2) |  |
| BMI 25-30 | 9 (0.3) | 20 (0.8) | 12 (0.8) |  |
| BMI >30 | 2583 (97.1) | 2527 (97.2) | 1514 (98.1) |  |
| Missing | 66 (2.5) | 48 (1.8) | 14 (0.9) |  |
|  |  |  |  |  |
| Comorbidities present n(%) | 767 (28.8) | 730 (28.1) | 468 (30.3) | **0.002** |
|  |  |  |  |  |
| Charlson Comorbidity Index (CCI) |  |  |  | **<0.001** |
| 0-1 | 714 (26.8) | 2 370 (91.2) | 1 353 (87.7) |  |
| 2+ | 94 (11.6) | 80 (3.3) | 81 (5.6) |  |
| Missing | 1 853 (69.6) | 149 (5.7) | 109 (7.1) |  |
|  |  |  |  |  |
| ASA grade n(%) |  |  |  | 0.06 |
| ASA grade 1-2 | 599 (22.5) | 518 (20.0) | 319 (20.7) |  |
| ASA grade 3-5 | 2 059 (77.5) | 2 078 (80.0) | 1 223 (79.3) |  |
|  |  |  |  |  |
| Perioperative SARS-Cov-2 infection n(%) |  |  | 1 (0.06) |  |
| Preoperative confirmed | - | - | 1 (0.06) |  |
| Postoperative confirmed | - | - | 1 (0.06) |  |
|  |  |  |  |  |
| Audit specific procedure n(%) |  |  |  | **<0.001** |
| Roux-en-Y Gastric Bypass | 1 509 (56.7) | 1 651 (63.5) | 991 (64.2) |  |
| Gastric Sleeve | 1 051 (39.5) | 836 (32.2) | 504 (32.7) |  |
| Other | 101 (3.8) | 112 (4.3) | 48 (3.1) |  |
| Abbreviations: BMI = Body Mass Index; ASA Score = American Society of Anesthesiologists Grade; | | | | |

**Tables S3a–S3h**

**Table S3a:** An overview of surgical planning of patients registered in the Surgical Dutch Lung Cancer Audit - (DLCA-S) undergoing surgery between 1^st^ January 2018 and the 31^st^ of December 2020.

| **Surgical planning DLCA-S** | **2018**  n=2 340 | **2019**  n=2 444 | **2020**  n=2 133 | **p-value** |
| --- | --- | --- | --- | --- |
| **Time to surgery** |  |  |  |  |
| Time to surgery (days) median [IQR] | 51.00 [36.00 74.00] | 51.00 [35.00 75.00] | 53.00 [35.00 77.00] | 0.88 |
| Oncologic | 51.00 [36.00 74.00] | 51.00 [35.00 75.00] | 53.00 [35.00 77.00] | 0.88 |
| Non-oncologic | NR* | NR* | NR* |  |
|  |  |  |  |  |
|  |  |  |  |  |
| Number of patients in additional survey | - | - | n=1 511 |  |
| Surgical treatment postponed n(%)  *(as indicated in additional survey)* | - | - | 67 (4.3) |  |
| Oncological | - | - | 24 (35.9) |  |
| Non-oncological | - | - | 43 (64.1) |  |
| Delay of surgery due to n(%): |  |  |  |  |
| Capacity | - | - | 38 (57.6) |  |
| (suspected) COVID-19 infection | - | - | 9 (14.5) |  |
| Other | - | - | 11 (17.7) |  |
|  |  |  |  |  |
| **Surgical treatment plan** |  |  |  |  |
| Used surgical approach n(%): |  |  |  | **0.003** |
| Minimally Invasive | 684 (29.2) | 702 (28.7) | 661 (31.0) |  |
| Open | 276 (11.8) | 223 (9.1) | 193 (9.1) |  |
| Unknown | 1 380 (58.9) | 1 519 (62.2) | 1 279 (60.0) |  |
|  |  |  |  |  |
|  |  |  |  |  |
| Number of patients in additional survey | - | - | n=1 502 |  |
| Surgical treatment plan changed n(%)  *(as indicated in additional survey)* | - | - | 0.0 (0.0) |  |
| Change of surgical approach due to: |  |  |  |  |
| Capacity | - | - | 0.0 (0.0) |  |
| COVID-19 infection | - | - | 0.0 (0.0) |  |
| Protection of the team | - | - | 0.0 (0.0) |  |
|  |  |  |  |  |
| **Neo-adjuvant treatment plan** |  |  |  | **0.009** |
| Receiving neo-adjuvant treatment n(%) | 94 (4.0) | 77 (3.2) | 108 (5.1) |  |
| Chemotherapy | 20 (0.9) | 24 (1.0) | 38 (1.8) |  |
| Radiotherapy | 5 (0.2) | 6 (0.2) | 3 (0.1) |  |
| Chemoradiotherapy |  |  |  |  |
| Number of patients in additional survey | - | - | n=1 499 |  |
| Neo-adjuvant treatment plan changed  *(as indicated in additional survey)* | - | - | 8 (0.5) |  |
| Other treatment | - | - | 2 (25.0) |  |
| Other scheme/dosing | - | - | 0(0.0) |  |
| No therapy | - | - | 5 (62.5) |  |
| Other | - | - | 1 (12.5) |  |
| *Time to surgery was not reported (NR) for non-oncologic surgical procedures in the DLCA-S Time to surgery is defined as number of days between date of first out-patient clinic visit and date of surgery. | | | | |

**Table S3b:** An overview of surgical planning of patients registered in the Dutch Upper GI Cancer Audit (DUCA) undergoing surgery between 1^st^ January 2018 and the 31^st^ of December 2020.

| **Surgical planning DUCA** | **2018**  n=830 | **2019**  n=811 | **2020**  n=931 | **p-value** |
| --- | --- | --- | --- | --- |
| **Time to surgery** |  |  |  |  |
| Time to surgery (days) median [IQR] | 114 [90.0 138 0] | 120 0 [97.0 146 0] | 113 [83.0 136 0] | **<0.001** |
| Oncologic | 114.00 [90.00 136.75] | 120.00 [95.00 143.00] | 112.00 [81.00 134.00] |  |
|  |  |  |  |  |
|  |  |  |  |  |
| Number of patients in additional survey |  |  | n=734 |  |
| Surgical treatment postponed n(%)  *(as indicated in additional survey)* | - | - | 23 (3.1) |  |
| Oncologic | - | - | 23 (100) |  |
| Non-oncologic | - | - | 0 (0) |  |
| Delay of surgery due to n(%): |  |  |  |  |
| Capacity | - | - | 6 (26.1) |  |
| (suspected) COVID-19 infection | - | - | 9 (39.1) |  |
| Other | - | - | 6 (26.1) |  |
|  |  |  |  |  |
| **Surgical treatment plan** |  |  |  |  |
| Used surgical approach n(%) |  |  |  |  |
| Minimally Invasive | 682 (82.2) | 692 (85.3) | 778 (83.6) | 0.23 |
| Open | 148 (17.8) | 117 (14.4) | 153 (16.4) |  |
| Unknown | 0 (0.0) | 2 (0.2) | 0 (0.0) |  |
|  |  |  |  |  |
| Number of patients in additional survey | - | - | n=737 |  |
| Surgical treatment plan changed n(%)  *(as indicated in additional survey)* | - | - | 2 (0.2) |  |
| Change of surgical approach due to: |  |  |  |  |
| Capacity | - | - | 2 (100.0) |  |
| COVID-19 infection | - | - | 0 (0.0) |  |
| Protection of the team | - | - | 0 (0.0) |  |
|  |  |  |  |  |
| **Neo-adjuvant treatment plan** |  |  |  |  |
| Receiving neo-adjuvant treatment n(%) | 676 (81.4) | 682 (84.1) | 751 (80.7) | 0.42 |
| Chemotherapy | 198 (23.9) | 214 (26.4) | 267 (28.7) | 0.13 |
| Radiotherapy | 2 (0.2) | 0 (0.0) | 1 (0.1) |  |
| Chemoradiotherapy | 476 (57.3) | 468 (57.7) | 483 (51.9) |  |
|  |  |  |  |  |
| Number of patients in additional survey | - | - | n=739 |  |
| Neo-adjuvant treatment plan changed n(%)  *(as indicated in additional survey)* | - | - | 11 (1.5) |  |
| Other treatment | - | - | 2 (18.2) |  |
| Other scheme/dosing | - | - | 4 (36.4) |  |
| No therapy | - | - | 5 (55.6) |  |
| Other | - | - | 0 (0.0) |  |
| Time to surgery is defined as number of days between date of first out-patient clinic visit and date of surgery | | | | |

**Table S3c**: An overview of surgical planning of patients registered in the Dutch Pancreatic Cancer Audit (DPCA) undergoing surgery between 1^st^ January 2018 and the 31^st^ of December 2020.

| **Surgical planning DPCA** | **2018**  n=626 | **2019**  n=636 | **2020**  n=663 | **p-value** |
| --- | --- | --- | --- | --- |
| **Time to surgery** |  |  |  |  |
| Time to surgery (days) median [IQR] | 34.00 [20.00 57.00] | 36.00 [22.00 75.00] | 39.00 [23.00 81.25] | **0.002** |
| Oncologic | 33.00 [20.00 54.75] | 35.00 [22.00 73.00] | 38.00 [23.00 77.75] | **<0.001** |
| Non-oncologic | 8.00 [40.00 105.00] | 50.00 [32.00 117.00] | 8.00 [33.75 132.00] |  |
|  |  |  |  |  |
| Number of patients in additional survey |  |  | n=503 |  |
| Surgical treatment postponed n(%)  *(as indicated in additional survey)* | - | - | 8 (1.6) |  |
| Oncologic | - | - | 6 (75) |  |
| Non-oncologic | - | - | 2 (25) |  |
| Delay of surgery due to n(%): |  |  |  |  |
| Capacity | - | - | 7 (87.5) |  |
| (suspected) COVID-19 infection | - | - | 1 (12.5) |  |
| Other | - | - | 0 (0) |  |
|  |  |  |  |  |
| **Surgical treatment plan** |  |  |  |  |
| Used surgical approach n(%): |  |  |  | 0.41 |
| Minimally Invasive | 109 (17.9) | 142 (22.8) | 137 (21.2) |  |
| Open | 472 (77.5) | 459 (73.8) | 483 (74.9) |  |
| Local treatment |  |  |  |  |
| Endovascular |  |  |  |  |
| Unknown | 28 (4.6) | 21 (3.4) | 25 (3.9) |  |
|  |  |  |  |  |
| Number of patients in additional survey |  |  | n=503 |  |
| Surgical treatment plan changed n(%)  *(as indicated in additional survey)* | - | - | 32 (4.8) |  |
| Change of surgical approach due to: |  |  |  |  |
| Capacity | - | - | 31 (96.9) |  |
| COVID-19 infection | - | - | 0 (0) |  |
| Protection of the team | - | - | 1 (3.1) |  |
| Other | - | - | 0 (0) |  |
|  |  |  |  |  |
| **Neo-adjuvant treatment plan** |  |  |  |  |
| Receiving neo-adjuvant treatment n(%) | 3 (1.1) | 26 (9.6) | 41 (13.7) | **<0.001** |
| Chemotherapy | 23 (3.8) | 50 (8.0) | 66 (10.2) | **<0.001** |
| Chemoradiotherapy | 2 (0.3) | 22 (3.5) | 33 (5.1) |  |
|  |  |  |  |  |
| Number of patients in additional survey |  |  | n=502 |  |
| Neo-adjuvant treatment plan changed n(%)  *(as indicated in additional survey)* | - | - | 3 (7.3) |  |
| Other treatment | - | - | 0 (0) |  |
| Other scheme/dosing | - | - | 1 (33.3) |  |
| No therapy | - | - | 1 (33.3) |  |
| Other | - | - | 1 (33.3) |  |
| Time to surgery is defined as number of days between date of first out-patient clinic visit and date of surgery | | | | |

**Table S3d:** An overview of surgical planning of patients registered in the Dutch Hepato Biliary Audit (DHBA) undergoing surgery between 1st January 2018 and the 31^st^ of December 2020.

| **Surgical planning DHBA** | **2018**  n=1 190 | **2019**  n=1 325 | **2020**  n=1 215 | **p-value** |
| --- | --- | --- | --- | --- |
| **Time to surgery** |  |  |  |  |
| Time to surgery (days) median [IQR] | 35.00 [21.00 57.00] | 35.00 [21.00 57.00] | 30.00 [19.00 49.00] | **<0.001** |
| Oncologic | 34.00 [21.00 55.00] | 34.00 [21.00 56.00] | 30.00 [19.00 48.00] | **<0.001** |
| Non-oncologic | 49.00 [29.00 87.00] | 50.00 [27.00 100.00] | 34.00 [16.00 74.00] |  |
|  |  |  |  |  |
| Number of patients in additional survey | - | - | n=841 |  |
| Surgical treatment postponed n(%)  *(as indicated in additional survey)* | - | - | 11 (1.3) |  |
| Oncologic | - | - | 6 (54.5) |  |
| Non-oncologic | - | - | 5 (45.5) |  |
| Delay of surgery due to n(%) |  |  |  |  |
| Capacity | - | - | 9 (81.8) |  |
| (suspected) COVID-19 infection | - | - | 0 (0.0) |  |
| Other | - | - | 2 (18.2) |  |
|  |  |  |  |  |
| **Surgical treatment plan** |  |  |  |  |
| Used surgical approach n(%) |  |  |  | **<0.001** |
| Minimally Invasive | 318 (26.7) | 327 (24.7) | 352 (29.0) |  |
| Open | 568 (47.7) | 528 (39.8) | 493 (40.6) |  |
| Local treatment | 175 (14.7) | 399 (30.1) | 310 (25.5) |  |
| Unknown | 129 (10.8) | 71 (5.4) | 60 (4.9) |  |
|  |  |  |  |  |
| Number of patients in additional survey | - | - | n=841 |  |
| Surgical treatment plan changed n(%)  *(as indicated in additional survey)* | - | - | 1 (0.08) |  |
| Change of surgical approach due to n(%) |  |  |  |  |
| Capacity | - | - | 1 (100) |  |
| COVID-19 infection | - | - | 0 |  |
| Protection of the team | - | - | 0 |  |
|  |  |  |  |  |
| **Neo-adjuvant treatment plan** |  |  |  |  |
| Receiving neo-adjuvant treatment n(%) | 237 (20.2) | 239 (18.0) | 226 (18.7) | **0.01** |
| Chemotherapy | 237 (100) | 239 (100) | 226 (100) | 0.39 |
|  |  |  |  |  |
| Number of patients in additional survey | - | - | n=845 |  |
| Neo-adjuvant treatment plan changed n(%)  *(as indicated in additional survey)* | - | - | 3 (0.3) |  |
| Other treatment | - | - | 0 (0) |  |
| Other scheme/dosing | - | - | 1 (33.3) |  |
| No therapy | - | - | 0 (0) |  |
| Other | - | - | 2 (66.7) |  |
| Time to surgery is defined as number of days between date of first out-patient clinic visit and date of surgery | | | | |

**Table S3e**: An overview of surgical planning of patients registered in the Dutch ColoRectal Audit (DCRA) undergoing surgery between 1^st^ January 2018 and the 31^st^ of December 2020.

| **Surgical planning overall DCRA** | **2018**  n=2 572 | **2019**  n=2 355 | **2020**  n=1 933 | **p-value** |
| --- | --- | --- | --- | --- |
| **Time to surgery** |  |  |  |  |
| Time to surgery (days) median [IQR] | 20.00 [10.00 33.00] | 19.00 [11.00 34.00] | 17.00 [8.00 34.00] | **0.01** |
|  |  |  |  |  |
| Number of patients in additional survey | - | - | n=848 |  |
| Surgical treatment postponed n(%)  *(as indicated in additional survey)* | - | - | 8 (0.9) |  |
| Oncologic | - | - | 7 (87.5) |  |
| Non-oncologic | - | - | 1 (12.5) |  |
| Delay of surgery due to n(%) |  |  |  |  |
| Capacity | - | - | 3 (37.5) |  |
| (suspected) COVID-19 infection | - | - | 3 (37.5) |  |
| Other | - | - | 3 (37.5) |  |
|  |  |  |  |  |
| **Surgical treatment plan** |  |  |  |  |
| Used surgical approach n(%) |  |  |  | **<0.001** |
| Minimally Invasive | 1 997 (77.7) | 1 993 (84.6) | 1 628 (84.2) |  |
| Open | 316 (12.3) | 257 (10.9) | 223 (11.5) |  |
| Local treatment | 73 (2.8) | 57 (2.4) | 40 (2.1) |  |
| Unknown | 185 (7.2) | 48 (2.0) | 42 (2.2) |  |
|  |  |  |  |  |
| Number of patients in additional survey | - | - | n=847 |  |
| Surgical treatment plan changed n(%)  *(as indicated in additional survey)* | - | - | 2 (0.2) |  |
| Change of surgical approach due to n(%) | - | - | 0 (0) |  |
| Capacity | - | - | 0 (0) |  |
| COVID-19 infection | - | - | 0 (0) |  |
| Protection of the team | - | - | 0 (0) |  |
|  |  |  |  |  |
| **Neo-adjuvant treatment plan** |  |  |  |  |
| Receiving neo-adjuvant treatment n(%) | 411 (16.0) | 333 (14.1) | 289 (15.0) | **0.02** |
| Chemotherapy | 196 (7.6) | 196 (8.3) | 145 (7.5) | **0.002** |
| Radiotherapy | 163 (6.3) | 109 (4.6) | 100 (5.2) |  |
| Chemoradiotherapy | 196 (7.6) | 196 (8.3) | 145 (7.5) |  |
|  |  |  |  |  |
| Number of patients in additional survey | - | - | n=844 |  |
| Neo-adjuvant treatment plan changed (%)  *(as indicated in additional survey)* | - | - | 1 (0.1) |  |
| Other treatment | - | - | 0 (0%) |  |
| Other scheme/dosing | - | - | 0 (0%) |  |
| No therapy | - | - | 0 (0%) |  |
| Other | - | - | 1 (100.0) |  |
| Time to surgery is defined as number of days between date of first out-patient clinic visit and date of surgery | | | | |

**Table S3f:** An overview of surgical planning of patients registered in the Dutch Hip Fracture Audit (DHFA) undergoing surgery between 1^st^ January 2018 and the 31^st^ of December 2020.

| **Surgical planning DHFA** | **2018**  n=1 994 | **2019**  n=2 276 | **2020**  n=2 138 | **p-value** |
| --- | --- | --- | --- | --- |
| **Time to surgery** |  |  |  |  |
| Time to surgery (days) median [IQR] | 1.00 [0.00 1.00] | 1.00 [0.00 1.00] | 1.00 [0.00 1.00] | 0.37 |
|  |  |  |  |  |
|  |  |  |  |  |
| Number of patients in additional survey | - | - | n=1 378 |  |
| Surgical treatment postponed n(%)  *(as indicated in additional survey)* | - | - | 31 (2.2) |  |
| Delay of surgery due to n(%) (n=829): |  |  |  |  |
| Capacity | - | - | 0 (0.0) |  |
| (suspected) COVID-19 infection | - | - | 23 (74.1) |  |
| Other | - | - | 10 (32.2) |  |
| Time to surgery is defined as number of days between date of emergency room visit and date of surgery | | | | |

**Table S3g**: An overview of surgical planning of patients registered in the Dutch Surgical Aneurysm Audit (DSAA) undergoing surgery between 1^st^ January 2018 and the 31^st^ of December 2020.

| **Surgical planning DSAA** | **2018**  n=1 772 | **2019**  n=1 761 | **2020**  n=1 595 | **p-value** |
| --- | --- | --- | --- | --- |
| Number of patients in additional survey |  |  | n=1360 |  |
| Surgical treatment postponed n(%)  *(as indicated in additional survey)* | - | - | 104 (7.6) |  |
| Delay of surgery due to n(%) |  |  |  |  |
| Capacity | - | - | 90 (86.5) |  |
| (suspected) COVID-19 infection | - | - | 4 (3.9) |  |
| Other | - | - | 13 (12.6) |  |
|  |  |  |  |  |
| **Surgical treatment plan** |  |  |  |  |
| Used surgical approach n(%): |  |  |  | 0.95 |
| Open | 538 (30.4) | 549 (31.2) | 482 (30.2) |  |
| Endovascular | 1214 (68.5) | 1214 (68.5) | 1214 (68.5) |  |
| Unknown | 20 (1.1) | 20 (1.1) | 20 (1.1) |  |
|  |  |  |  |  |
| Number of patients in additional survey | - | - | n=1 376 |  |
| Surgical treatment plan changed n(%) *(as indicated in additional survey* (n(%)) | - | - | 0 (0.0) |  |
| Time to surgery is defined as number of days between date of first out-patient clinic visit and date of surgery | | | | |

**Table S3h**: An overview of surgical planning of patients registered in the Dutch Audit for Treatment of Obesity (DATO) undergoing surgery between 1^st^ January 2018 and the 31^st^ of December 2020.

| **Surgical planning DATO** | **2018**  n=2 661 | **2019**  n=2 599 | **2020**  n=1 543 | **p-value** |
| --- | --- | --- | --- | --- |
| **Time to surgery** |  |  |  |  |
| Time to surgery (days) median [IQR] | 158.00 [112.00 256.00] | 139.00 [89.00 247.75] | 190.00 [126.00 279.00] | **<0.001** |
|  |  |  |  |  |
|  |  |  |  |  |
| Number of patients in additional survey | - | - | n=1 533 |  |
| Surgical treatment postponed n(%)  *(as indicated in additional survey)* | - | - | 587 (38.2) |  |
| Delay of surgery due to n(%): | - | - |  |  |
| Capacity | - | - | 564 (96.2) |  |
| (suspected) COVID-19 infection | - | - | 4 (0.7) |  |
| Other | - | - | 2 (0.3) |  |
|  |  |  |  |  |
| **Surgical treatment plan** |  |  |  |  |
| Used surgical approach n(%) |  |  |  | 0.84 |
| Minimally Invasive | 2 658 (99.9) | 2 593 (99.9) | 1 541 (99.9) |  |
| Open | 2 (0.1) | 3 (0.1) | 2 (0.1) |  |
|  |  |  |  |  |
|  |  |  |  |  |
| Number of patients in additional survey | - | - | n=1 533 |  |
| Surgical treatment plan changed n(%)  *(as indicated in additional survey)* | - | - | 114 (7.4) |  |
| Change of surgical approach due to: | - | - |  |  |
| Capacity | - | - | 110 (96.5) |  |
| COVID-19 infection | - | - | 0 (0) |  |
| Protection of the team | - | - | 1 (0.9) |  |
| Time to surgery is defined as number of days between date of first out-patient clinic visit and date of surgery | | | | |

**Tables S4a–S4h**

**Table S4a:** An overview of surgical outcomes during the first COVID-19 wave, the second COVID-19 wave and the interim period of the year 2020 compared with historical reference cohorts of patients registered in the Surgical Dutch Lung Cancer Audit - (DLCA-S) undergoing surgery between 1^st^ January 2018 and the 31^st^ of December 2020.

| **Surgical outcomes DLCA-S** | **First wave**  n=371 | **Reference first wave**  n=917 | **p-value** | **Second wave**  n=583 | **Reference 2nd wave**  n=1 189 | **p-value** | **Interim period**  n=667 | **Reference interim period** n=1 655 | **p-value** |
| --- | --- | --- | --- | --- | --- | --- | --- | --- | --- |
| Length of hospital stay median [IQR] | 4.00 [2.00 6.00] | 5.00 [2.00 7.00] | **0.01** | 4.00 [2.00 6.00] | 5.00 [3.00 7.00] | **<0.001** | 4.00 [2.00 7.00] | 4.00 [2.00 7.00] | 0.37 |
| Severe complication * n(%) | 26 (7.0) | 94 (10.3) | 0.08 | 56 (9.6) | 94 (7.9) | 0.26 | 58 (8.7) | 162 (9.8) | 0.26 |
| Readmission within 30 days n(%) | 15 (4.0) | 51 (5.6) | 0.39 | 26 (4.5) | 74 (6.2) | 0.27 | 40 (6.0) | 64 (3.9) | 0.08 |
| 30-day mortality n(%) | 4 (1.1) | 23 (2.5) | **0.03** | 14 (2.4) | 16 (1.3) | 0.22 | 11 (1.6) | 37 (2.2) | 0.57 |
| ICU admission n(%) | 76 (20.5) | 197 (21.5) | **0.05** | 91 (15.6) | 199 16.7) | **<0.001** | 106 (15.9) | 308 (18.6) | **<0.001** |
| Length of ICU admission median [IQR] | 0.00 [0.00 0.00] | 0.00 [0.00 0.00] | 0.46 | 0.00 [0.00 0.00] | 0.00 [0.00 0.00] | 0.07 | 0.00 [0.00 0.00] | 0.00 [0.00 0.00] | **0.006** |
| COVID related ICU admission n(%) | 0.0 (0.0) | - |  | 4 (0.7) | - |  | 0.0 (0.0) | - |  |
|  |  |  |  |  |  |  |  |  |  |
| **Oncological details** |  |  |  |  |  |  |  |  |  |
| Resection margins n(%) |  |  | 0.07 |  |  | **0.05** |  |  | 0.11 |
| R0 | 158 (76.3) | 333 (74.0) |  | 230 (72.6) | 415 (78.2) |  | 213 (69.8) | 644 (76.3) |  |
| R1 | 1 (0.5) | 15 (3.3) |  | 8 (2.5) | 17 (3.2) |  | 8 (2.6) | 19 (2.3) |  |
| R2 | 1 (0.5) | 0 (0.0) |  | 2 (0.6) | 0 (0.0) |  | 0 (0.0) | 2 (0.2) |  |
| Unknown | 47 (22.7) | 102 (22.7) |  | 77 (24.3) | 99 (18.6) |  | 84 (27.5) | 179 (21.2) |  |
| Clinical TNM stage n(%) |  |  | 0.87 |  |  | **0.001** |  |  | **0.04** |
| 0 | 8 (3.9) | 12 (2.7) |  | 13 (4.1) | 15 (2.8) |  | 7 (2.3) | 17 (2.0) |  |
| I | 67 (32.4) | 167 (37.1) |  | 98 (30.9) | 192 (36.2) |  | 91 (29.8) | 294 (34.8) |  |
| II | 42 (20.3) | 80 (17.8) |  | 53 (16.7) | 106 (20.0) |  | 53 (17.4) | 183 (21.7) |  |
| III | 25 (12.1) | 52 (11.6) |  | 39 (12.3) | 92 (17.3) |  | 38 (12.5) | 115 (13.6) |  |
| IV | 4 (1.9) | 9 (2.0) |  | 8 (2.5) | 9 (1.7) |  | 11 (3.6) | 18 (2.1) |  |
| X | 18 (8.7) | 33 (7.3) |  | 32 (10.1) | 22 (4.1) |  | 29 (9.5) | 49 (5.8) |  |
| Missing | 43 (20.8) | 97 (21.6) |  | 74 (23.3) | 95 (17.9) |  | 76 (24.9) | 1. 9.9) |  |
| *Severe complications defined as need for reintervention, ICU-admission and/or death, Length of hospital stay is the number of days between date of surgery and date of discharge. Abbreviations: ICU = Intensive Care Unit. | | | | | | | | | |

**Table S4b:** An overview of surgical outcomes during the first COVID-19 wave, the second COVID-19 wave and the interim period of the year 2020 compared with historical reference cohorts of patients registered in the Dutch Upper Gastrointestinal Cancer Audit (DUCA) undergoing surgery between 1^st^ January 2018 and the 31^st^ of December 2020.

| **Surgical outcomes DUCA** | **First wave**  n=208 | **Reference first wave**  n= 301 | **p-value** | **Second wave**  n= 264 | **Reference 2nd wave**  n= 463 | **p-value** | **Interim period**  n= 264 | **Reference interim period**  n= 526 | **p-value** |
| --- | --- | --- | --- | --- | --- | --- | --- | --- | --- |
| Length of hospital stay median [IQR] | 8.00 [6.00 12.00] | 10.00 [7.00 16.00] | **<0.001** | 8.00 [6.00 13.25] | 9.00 [7.00 14.00] | **0.09** | 9.00 [7.00 14.00] | 9.00 [7.00 14.00] | 0.22 |
| Severe complication * n(%) | 55 (26.4) | 89 (29.6) | 0.50 | 63 (23.9) | 110 (23.8) | 1.00 | 70 (26.5) | 129 (24.5) | 0.60 |
| Readmission within 30 days n(%) | 35 (16.8) | 41 (13.6) | 0.58 | 49 (18.6) | 60 (13.0) | **0.02** | 46 (17.4) | 78 (14.8) | 0.63 |
| 30-day mortality n(%) | 7 (3.4) | 17 (5.6) | 0.32 | 12 (4.5) | 14 (3.0) | 0.39 | 9 (3.4) | 15 (2.9) | 0.83 |
| ICU admission n(%) | 107 (51.4) | 212 (70.4) | **<0.001** | 149 (56.4) | 317 (68.5) | **0.002** | 148 (56.1) | 367 (69.8) | **<0.001** |
| Length of ICU admission median [IQR] | 1.00 [0.00 1.00] | 1.00 [0.00 3.00] | **<0.001** | 1.00 [0.00 1.00] | 1.00 [0.00 2.00] | **<0.001** | 1.00 [0.00 2.00] | 1.00 [0.00 2.00] | **0.001** |
|  |  |  |  |  |  |  |  |  |  |
| **Oncological details** |  |  |  |  |  |  |  |  |  |
| Resection margins n(%) |  |  | 0.16 |  |  | 0.54 |  |  | 0.44 |
| R0 | 186 (89.4) | 253 (84.1) |  | 232 (87.9) | 396 (85.5) |  | 233 (88.3) | 449 (85.4) |  |
| R1 | 12 (5.8) | 18 (6.0) |  | 14 (5.3) | 24 (5.2) |  | 16 (6.1) | 30 (5.7) |  |
| R2 | 1 (0.5) | 1 (0.3) |  | 0 (0.0) | 2 (0.4) |  | 1 (0.4) | 2 (0.4) |  |
| Clinical TNM stage n(%) |  |  | 0.97 |  |  | **0.001** |  |  | **0.02** |
| 0 | 34 (16.3) | 48 (15.9) |  | 25 (9.5) | 65 (14.0) |  | 25 (9.5) | 84 (16.0) |  |
| I | 40 (19.2) | 58 (19.3) |  | 53 (20.1) | 116 (25.1) |  | 59 (22.3) | 126 (24.0) |  |
| II | 51 (24.5) | 66 (21.9) |  | 61 (23.1) | 113 (24.4) |  | 62 (23.5) | 138 (26.2) |  |
| III | 60 (28.8) | 94 (31.2) |  | 85 (32.2) | 114 (24.6) |  | 76 (28.8) | 114 (21.7) |  |
| IV | 6 (2.9) | 7 (2.3) |  | 4 (1.5) | 23 (5.0) |  | 10 (3.8) | 25 (4.8) |  |
| X | 2 (1.0) | 5 (1.7) |  | 10 (3.8) | 6 (1.3) |  | 7 (2.7) | 28 (5.3) |  |
| Missing | 15 (7.2) | 23 (7.6) |  | 26 (9.8) | 26 (5.6) |  | 25 (9.5) | 11 (2.1) |  |
| *Severe complications defined as need for reintervention, ICU-admission and/or death, Length of hospital stay is the number of days between date of surgery and date of discharge. Abbreviations: ICU = Intensive Care Unit. | | | | | | | | | |

**Table S4c**: An overview of surgical outcomes during the first COVID-19 wave, the second COVID-19 wave and the interim period of the year 2020 compared with historical reference cohorts of patients registered in the Dutch Pancreatic Cancer Audit (DPCA) undergoing surgery between 1^st^ January 2018 and the 31^st^ of December 2020.

| **Surgical outcomes DPCA** | **First wave**  N=131 | **Reference first wave**  n=238 | **p-value** | **Second wave**  n=191 | **Reference 2nd wave**  n=360 | **p-value** | **Interim period**  n=205 | **Reference interim period** n=406 | **p-value** |
| --- | --- | --- | --- | --- | --- | --- | --- | --- | --- |
| Length of hospital stay median [IQR] | 9.00 [6.00 13.75] | 9.00 [7.00 16.00] | 0.34 | 9.50 [7.00 16.00] | 10.00 [7.00 16.00] | 0.61 | 9.00 [7.00 15.00] | 10.00 [7.00 16.00] | 0.17 |
| Severe complication *n(%) | 31 (25.0) | 56 (25.3) | 0.20 | 60 (31.4) | 91 (25.3) | **0.03** | 56 (27.3) | 127 (31.3) | **0.01** |
| Readmission within 30 days n(%) | 36 (27.5) | 63 (26.5) | 0.58 | 25 (13.1) | 45 (12.5) | 0.19 | 32 (15.6) | 52 (12.8) | 0.57 |
| 30-day mortality n(%) | 3 (2.3) | 7 (2.9) | 0.97 | 5 (2.6) | 2 (0.6) | 0.09 | 6 (2.9) | 7 (1.7) | 0.22 |
| ICU admission n(%) | 11 (8.4) | 21 (8.8) | 0.32 | 12 (6.3) | 18 (5.0) | 0.75 | 7 (3.4) | 27 (6.7) | 0.19 |
| COVID related ICU admission n(%) | 0.0 (0.0) | - |  | 0.0 (0.0) | - |  | 0.0 (0.0) | - |  |
|  |  |  |  |  |  |  |  |  |  |
| **Oncologic** |  |  |  |  |  |  |  |  |  |
| Resection margins n(%) |  |  | **0.04** |  |  | 0.06 |  |  | 0.51 |
| R0 | 32 (24.4) | 41 (17.2) |  | 33 (17.3) | 65 (18.1) |  | 41 (20.0) | 64 (15.8) |  |
| R1 | 29 (22.1) | 40 (16.8) |  | 36 (18.8) | 43 (11.9) |  | 27 (13.2) | 66 (16.3) |  |
| R2 | 0 | 0 |  | 0 | 1 (0.3) |  | 1 (0.5) | 0 |  |
| Unknown | 13 (9.9) | 16 (6.7) |  | 14 (7.3) | 45 (12.5) |  | 17 (8.3) | 34 (8.4) |  |
| TNM stage of n(%)  Pancreatic ductal adenocarcinoma (PDAC) |  |  | **0.03** |  |  | 0.51 |  |  | 0.05 |
| I | 26 (19.8) | 31 (13.0) |  | 22 (11.5) | 42 (11.7) |  | 27 (13.2) | 49 (12.1) |  |
| II | 30 (22.9) | 34 (14.3) |  | 28 (14.7) | 45 (12.5) |  | 36 (17.6) | 56 (13.8) |  |
| III | 18 (13.7) | 50 (21.0) |  | 30 (15.7) | 67 (18.6) |  | 25 (12.2) | 89 (21.9) |  |
| IV | 0 (0.0) | 1 (0.4) |  | 0 (0.0) | 4 (1.1) |  | 0 (0.0) | 1 (0.2) |  |
| *Severe complications defined as need for reintervention, ICU-admission and/or death, Length of hospital stay is the number of days between date of surgery and date of discharge. Abbreviations: ICU = Intensive Care Unit. | | | | | | | | | |

**Table S4d:** An overview of surgical outcomes during the first COVID-19 wave, the second COVID-19 wave and the interim period of the year 2020 compared with historical reference cohorts of patients registered in the Dutch Hepato Biliary Audit (DHBA) undergoing surgery between 1^st^ January 2018 and the 31^st^ of December 2020.

| **Surgical outcomes DHBA** | **First wave**  n=244 | **Reference first wave**  n=478 | **p-value** | **Second wave**  n=315 | **Reference second wave**  n=699 | **p- value** | **Interim period**  n=400 | **Reference interim period** n=802 | **p-value** |
| --- | --- | --- | --- | --- | --- | --- | --- | --- | --- |
| Length of hospital stay median [IQR] | 4.00 [1.00 7.00] | 5.00 [1.00 7.00] | 0.14 | 4.00 [1.00 7.00] | 4.00 [1.00 7.00] | 0.38 | 4.00 [1.00 7.00] | 4.00 [2.00 7.00] | **0.03** |
| Severe complication *n(%) | 32 (13.1) | 49 (10.3) | 0.32 | 36 (11.4) | 64 (9.2) | 0.33 | 44 (11.0) | 83 (10.3) | 0.80 |
| Readmission within 30 days n(%) ^#^ | 14 (5.7) | 28 (11.0) | **0.05** | 27 (8.7) | 43 (11.5) | 0.41 | 40 (10) | 34 (8.1) | 0.12 |
| 30-day mortality n(%) | 1 (0.4) | 11 (2.3) | 0.11 | 5 (1.6) | 15 (2.1) | 0.51 | 3 (0.8) | 12 (1.5) | 0.19 |
| ICU admission n(%) | 33 (13.5) | 66 (13.8) | 0.27 | 38 (12.1) | 110 (15.7) | 0.19 | 63 (15.8) | 113 (14.1) | 0.35 |
| Length of ICU admission median [IQR] | 0.00 [0.00 0.00] | 0.00 [0.00 0.00] | 0.691 | 0.00 [0.00 0.00] | 0.00 [0.00 0.00] | 0.15 | 0.00 [0.00 0.00] | 0.00 [0.00 0.00] | 0.63 |
|  |  |  |  |  |  |  |  |  |  |
| **Oncological details** |  |  |  |  |  |  |  |  |  |
| Resection margins n(%) |  |  | 0.07 |  |  | 0.98 |  |  | 0.11 |
| R0 | 123 (50.4) | 221 (46.2) |  | 154 (48.9) | 338 (48.4) |  | 188 (47.0) | 419 (52.2) |  |
| R1 | 28 (11.5) | 39 (8.2) |  | 31 (9.8) | 65 (9.3) |  | 36 (9.0) | 58 (7.2) |  |
| R2 | 4 (1.6) | 3 (0.6) |  | 3 (1.0) | 6 (0.9) |  | 0 (0.0) | 5 (0.6) |  |
| Unknown | 89 (36.5) | 215 (45.0) |  | 127 (40.3) | 290 (41.5) |  | 176 (44.0) | 320 (39.9) |  |
| *Severe complications defined as need for reintervention, ICU-admission and/or death, # Readmission rate has been registered in the clinical audit since 2019. Therefore the comparison with the reference groups of the first wave second wave and interim period are calculated based on 2019 only (total of 255 374 and 420 respectively)  Length of hospital stay is the number of days between date of surgery and date of discharge. Abbreviations: ICU = Intensive Care Unit. | | | | | | | | | |

**Table S4e**: An overview of surgical outcomes during the first COVID-19 wave, the second COVID-19 wave and the interim period of the year 2020 compared with historical reference cohorts of patients registered in the Dutch Colo Rectal Audit (DCRA) undergoing surgery between 1^st^ January 2018 and the 31^st^ of December 2020.

| **Surgical outcomes DCRA** | **First wave**  n=342 | **Reference first wave**  n=948 | **p-value** | **Second wave**  n=549 | **Reference 2nd wave**  n=1 338 | **p-value** | **Interim period**  n=606 | **Reference interim**  **period** n=1 591 | **p-value** |
| --- | --- | --- | --- | --- | --- | --- | --- | --- | --- |
| Length of hospital stay median [IQR] | 4.00 [3.00 7.00] | 5.00 [3.00 7.00] | **0.08** | 4.00 [3.00 8.00] | 4.00 [3.00 8.00] | **0.07** | 4.00 [3.00 7.00] | 5.00 [3.00 8.00] | **0.01** |
| Severe complication * n(%) | 38 (11.1) | 141 (14.9) | 0.10 | 74 (13.5) | 195 (14.6) | 0.58 | 93 (15.3) | 265 (16.7) | 0.49 |
| Readmission within 30 days n(%) | 32 (9.4) | 110 (11.6) | 0.30 | 36 (6.6) | 110 (8.2) | **<0.001** | 54 (8.9) | 180 (11.3) | 0.14 |
| 90-day mortality n(%) | 8 (2.3) | 20 (2.1) | 0.97 | 45 (8.2) | 50 (3.7) | **<0.001** | 19 (3.1) | 30 (1.9) | 0.10 |
| ICU admission n(%) | 17 (5.0) | 83 (8.8) | **0.08** | 35 (6.4) | 122 (9.1) | **0.009** | 52 (8.6) | 159 (10.0) | **0.01** |
| Length of ICU admission median [IQR] | 3.00 [2.00 4.00] | 2.00 [1.00 4.00] | 0.16 | 2.00 [1.00 3.50] | 2.00 [1.00 4.00] | 0.51 | 2.00 [1.00 4.25] | 2.00 [1.00 4.00] | 0.67 |
|  |  |  |  |  |  |  |  |  |  |
| **Oncological details** |  |  |  |  |  |  |  |  |  |
| Resection margins n(%) |  |  | 0.10 |  |  | 0.07 |  |  | **0.004** |
| R0 | 326 (95.3) | 924 (97.5) |  | 520 (94.7) | 1 288 (96.3) |  | 574 (94.7) | 1 544 (97.0) |  |
| R1 | 11 (3.2) | 12 (1.3) |  | 19 (3.5) | 21 (1.6) |  | 21 (3.5) | 24 (1.5) |  |
| R2 | 1 (0.3) | 1 (0.1) |  | 2 (0.4) | 6 (0.4) |  | 4 (0.7) | 2 (0.1) |  |
| Unknown | 4 (1.2) | 11 (1.2) |  | 0 (0.0) | 0 (0.0 |  | 7 (1.2) | 21 (1.3) |  |
| Clinical TNM stage n(%) |  |  | 0.55 |  |  | 0.30 |  |  | **0.001** |
| I | 87 (26.2) | 245(27.7) |  | 136 (25.0) | 358 (28.4) |  | 135 (22.7) | 450 (29.0) |  |
| II | 111 (33.4) | 258 (29.2) |  | 182 (33.5) | 393 (31.2) |  | 205 (34.4) | 454 (30.2) |  |
| III | 109 (32.8) | 313 (35.4) |  | 195 (35.9) | 425 (33.7) |  | 209 (35.1) | 524 (34.9) |  |
| IV | 25 (7.5) | 67 (7.6) |  | 30 (5.5) | 85 (6.7) |  | 47 (7.9) | 75 (5.0) |  |
| *Severe complications defined as need for reintervention, ICU-admission and/or death, Length of hospital stay is the number of days between date of surgery and date of discharge. Abbreviations: ICU = Intensive Care Unit. | | | | | | | | | |

**Table S4f:** An overview of surgical outcomes during the first COVID-19 wave, the second COVID-19 wave and the interim period = of the year 2020 compared with historical reference cohorts of patients registered in the Dutch Hip Fracture Audit (DHFA) undergoing surgery between 1^st^ January 2018 and the 31^st^ of December 2020.

| **Surgical outcomes DHFA** | **First wave**  n= 380 | **Reference first wave**  n= 840 | **p-value** | **Second wave**  n= 566 | **Reference 2nd wave**  n= 1 115 | **p-value** | **Interim period**  n= 715 | **Reference interim period** n= 1 310 | **p-value** |
| --- | --- | --- | --- | --- | --- | --- | --- | --- | --- |
| Length of hospital stay median [IQR] | 5.00 [3.00 7.00] | 6.00 [4.00 10.00] | **<0.001** | 5.00 [3.00 8.00] | 6.00 [3.00 10.00] | 0.07 | 5.00 [3.00 7.00] | 6.00 [4.00 9.00] | **<0.001** |
| 30-day mortality n(%) | 15 (3.9) | 41 (4.9) | 0.56 | 30 (5.3) | 55 (4.9) | 0.83 | 24 (3.4) | 57 (4.4) | 0.33 |
| Length of hospital stay is the number of days between date of surgery and date of discharge. | | | | | | | | | |

**Table S4g**: An overview of surgical outcomes during the first COVID-19 wave, the second COVID-19 wave and the interim period of the year 2020 compared with historical reference cohorts of patients registered in the Dutch Surgical Aneurysm Audit (DSAA) undergoing surgery between 1^st^ January 2018 and the 31^st^ of December 2020.

| **Surgical outcomes DSAA** | **First wave**  n= 221 | **Reference first wave**  n= 685 | **p-value** | **Second wave**  n= 481 | **Reference 2nd wave**  n= 920 | **p-value** | **Interim period**  n= 501 | **Reference interim period** n= 1 127 | **p-value** |
| --- | --- | --- | --- | --- | --- | --- | --- | --- | --- |
| Length of hospital stay median [IQR] | 2.50 [1.00 7.00] | 4.00 [2.00 8.00] | **0.01** | 3.00 [1.00 7.00] | 4.00 [2.00 8.00] | **0.002** | 3.00 [1.25 8.00] | 3.00 [2.00 8.00] | 0.20 |
| Readmission within 30 days | 14 (6.3) | 60 (8.8) | 0.31 | 24 (5.0) | 49 (5.3) | 0.69 | 29 (5.8) | 77 (6.8) | 0.66 |
| 30-day mortality n(%) | 19 (8.6) | 45 (6.6) | 0.12 | 25 (5.2) | 75 (8.2) | 0.07 | 30 (6.0) | 68 (6.0) | 0.70 |
| ICU admission n(%) | 110 (49.8) | 335 (48.9) | 0.88 | 204 (42.4) | 468 (50.9) | **0.005** | 237 (47.3) | 531 (47.1) | 0.83 |
| Length of ICU admissio median [IQR] | 0.00 [0.00 1.00] | 0.00 [0.00 2.00] | 0.75 | 0.00 [0.00 1.00] | 0.00 [0.00 2.00] | **0.01** | 0.00 [0.00 1.00] | 0.00 [0.00 2.00] | 0.68 |
| Length of hospital stay is the number of days between date of surgery and date of discharge. Abbreviations: ICU = Intensive Care Unit. | | | | | | | | | |

**Table S4h**: An overview of surgical outcomes during the first COVID-19 wave, the second COVID-19 wave and the interim period of the year 2020 compared with historical reference cohorts of patients registered in the Dutch Audit for Treatment of Obesity (DATO) undergoing surgery between 1^st^ January 2018 and the 31^st^ of December 2020.

| **Surgical outcomes DATO** | **First wave**  n=21 | **Reference first wave**  n=1 023 | **p-value** | **Second wave**  n=361 | **Reference second wave**  n=1 453 | **p-value** | **Interim period**  n= 662 | **Reference interim period**  n= 1 523 | **p-value** |
| --- | --- | --- | --- | --- | --- | --- | --- | --- | --- |
| Length of hospital stay median [IQR] | 1.00 [1.00 1.00] | 1.00 [1.00 1.00] | 0.22 | 1.00 [1.00 1.00] | 1.00 [1.00 1.00] | 0.73 | 1.00 [1.00 1.00] | 1.00 [1.00 1.00] | 0.30 |
| Severe complication* | 0 (0.0) | 22 (2.2) | 1.00 | 11 (3.0) | 35 (2.4) | 0.61 | 16 (2.4) | 29 (1.9) | 0.54 |
| Readmission within 30 days | 1 (4.8) | 22 (2.2) | 0.53 | 12 (3.3) | 50 (3.4) | 0.92 | 25 (3.8) | 45 (3.0) | 0.30 |
| 30-day mortality n(%) | 0 (0.0) | 0 (0.0) | 1.0 | 0 (0.0) | 0 (0.0) | 1.0 | 0 (0.0) | 1 (0.1) | 1.0 |
| ICU admission n(%) | 0 (0.0) | 4 (0.4) | 0.96 | 1 (0.3) | 11 (0.8) | 0.60 | 4 (0.6) | 11 (0.7) | 0.94 |
| Length of ICU admission median [IQR] | NA [NA NA] | 1.00 [0.75 1.50] | - | 3.00 [3.00 3.00] | 1.00 [1.00 11.50] | 0.65 | 11.50 [2.50 22.75] | 2.00 [1.50 4.50] | 0.25 |
| *Severe complications defined as need for reintervention, ICU-admission and/or death, Length of hospital stay is the number of days between date of surgery and date of discharge. Abbreviations: ICU = Intensive Care Unit. | | | | | | | | | |

**Appendix S3 - Supplementary Figures**

**Figure S1.**

**
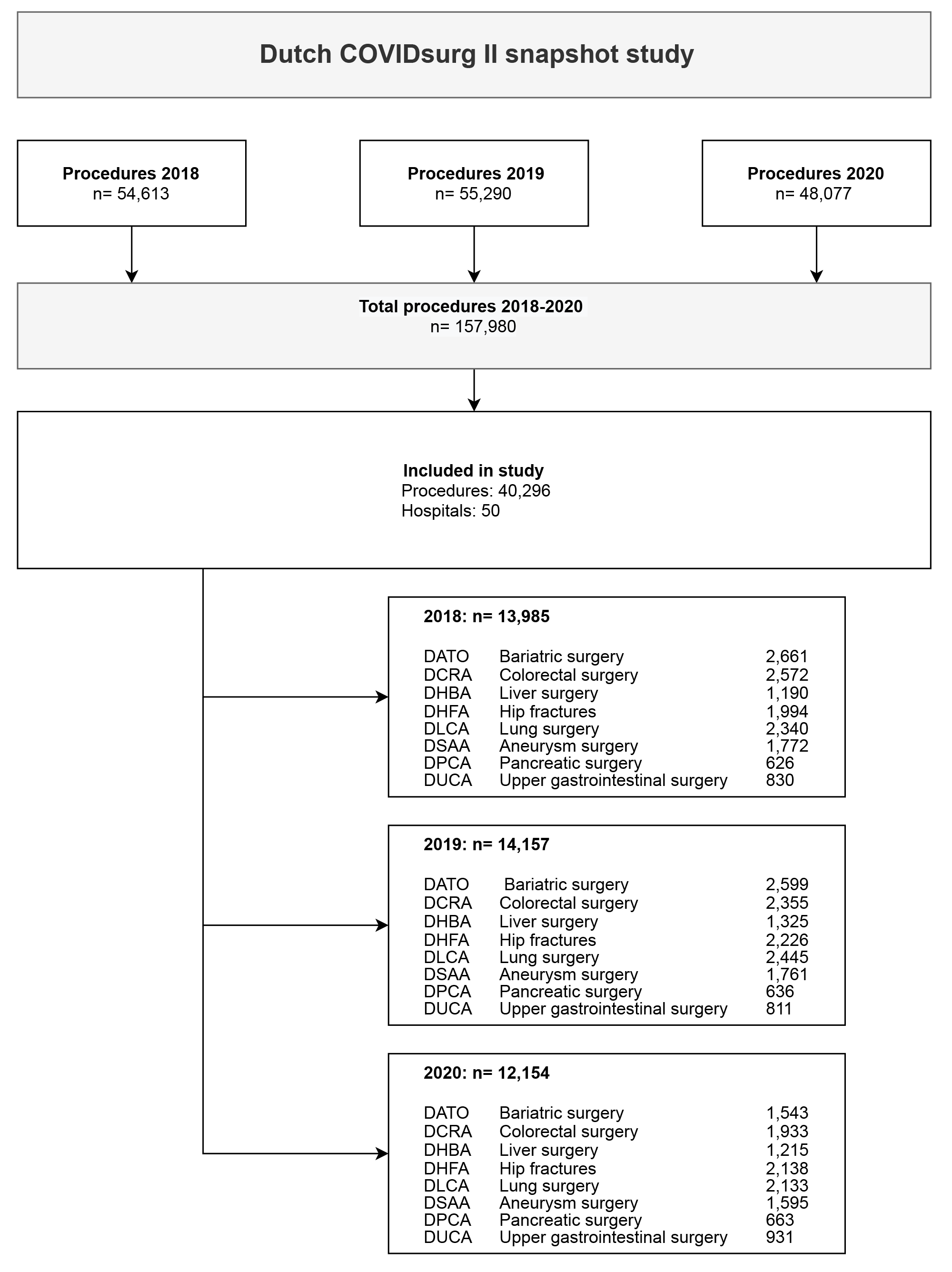
**

**Figure S1.**

**Title:** Flowchart of inclusion of participating hospitals and operated patients per surgical audit in the Dutch COVIDSurg II Snapshot Study

**Figure S2a–S2h**

**
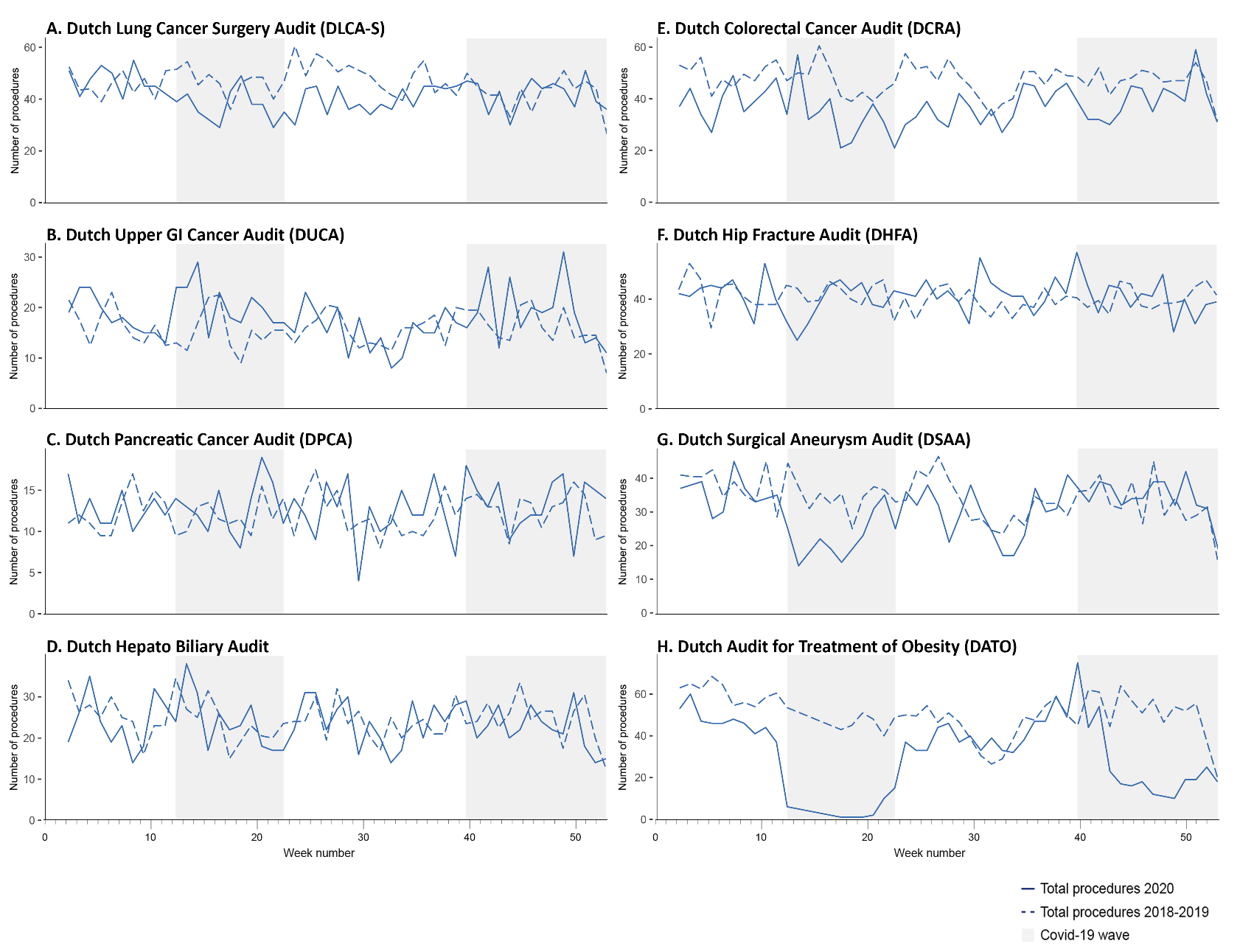
**

**Figure S2.** A graphical representation showing patients who were operated on every week during 2020 compared to the mean procedures performed in the historical reference cohort of 2018/2019. The periods of the COVID-19 waves are marked with a grey background. The dashed-line indicates the total number of procedures during 2018-2019, the solid-line indicates the total number of procedures during 2020.

**A.** Total number of performed lung cancer procedures; **B.** Total number of performed upper gastrointestinal cancer procedures; **C.** Total number of performed pancreatic cancer procedures; **D.** Total number of performed hepatobiliary procedures; **E.** Total number of performed colorectal cancer procedures; **F.** Total number of performed hip fracture procedures; **G.** Total number of performed aortic aneurysm procedures; **H.** Total number of performed bariatric procedures.

**Appendix S4: Case Report Form COVIDSurg snapshot study**

*The additional COVID-10 survey, translated in English*

**CASE REPORT FORM COVID-SURG-II SNAPSHOT STUDY – ENGLISH TRANSLATED VERSION**

| Patient ID |  |
| --- | --- |

**COVID SNAPSHOT MODULE**

| 1. Was there a peri-operative clinical suspicion or positive test a for SARS-CoV-2 (COVID-19) infection in this patient? | | - Yes - No |
| --- | --- | --- |
| **Impact of COVID-19 on treatment (NB! NOT related to pre-operative COVID-19 status of the patient)** | | |
| 1. Did COVID-19 have impact on (neoadjuvant) treatment? | | - Yes - No - Unknown |
| If YES on question 2. | | |
| 1. What was the impact on neoadjuvant treatment? | | - Neoadjuvant treatment has been waived - Type of neoadjuvant treatment has changed (for example: chemo-radiation instead of chemotherapy) - Dose or interval of neoadjuvant regimes has changed - Type of drug has changed (for example 5FU instead of docetaxel) - Other |
| 1. Did COVID-19 have impact on surgical planning? Or was surgery delayed? | | - Yes - No - Unknown |
| 1. What was the reason for impact on surgical planning or delay of surgery?* | | - Due to a (suspected) COVID-19 infection - Due to lack of health care capacity - Other |
| 1. Did COVID-19 have impact on surgical approach? (laparoscopic/robot assisted/open) | | - Yes - No - Unknown |
| If YES on question 6. | | |
| 1. What was the reason for the impact on surgical approach? (laparoscopic/robot assisted/open) | | - Due to a (suspected) COVID-19 infection - Due to lack of capacity - Because of protection of the surgical team - Other |
| 1. Does the patient have a history of smoking? | - Yes, current smoker - No, never smoked - Yes, currently stopped, but has a history of smoking. | |
| 1. Total number of packyears |  | |
| 1. What is the patient’s height? (in cm) |  | |
| 1. What is the patient’s weight? (in kg) |  | |

**If NO on question 1, this is the end of the additional COVID-19 survey**

**If YES on question 1, please fill in the additional questions below**

**Preoperative**

| 1. Did the patient experience symptoms related to COVID-19 preoperatively? | - Yes - No - Unknown |
| --- | --- |
| If No/Unknown, proceed to question **15** | |
| If YES | |
| 1. Which symptoms? | - Cough - Fever - Dypnea - Loss of smell and taste - Other |
| 1. Number of days before surgery, where the patient experienced symptoms which could have been related to COVID-19 |  |

**Preoperative COVID tests**

| 1. Was the patient tested on COVID-19 preoperatively? | - Yes - No - Unknown |
| --- | --- |
| If No/Unknown, proceed to question **25** | |
| If YES | |
| 1. Which type of tests did the patient undergo preoperatively? | - RT-PCR - Thoracix X-ray or CT - Serology - Other |
| If RT-PCR | |
| 1. Number of times the RT-PCR test was performed preoperatively |  |
| 1. What was the date of the first preoperative RT-PCR test? |  |
| 1. What was the date of the first preoperative positive RT-PCR test? |  |
| If thoracic X-ray of CT | |
| 1. Thoracic X-ray: Where consolidations suspected for COVID-19 visible? | - Not performed - Not suspected - Unilateral - Bilateral - Unknown |
| 1. Thoracic CT-scan – What was the CORADS score? | - Not performed - 1 - 2 - 3 - 4 - 5 - Unknown |
| 1. Number of Thoracic CT-scans made for a (suspected) COVID-19 infection? |  |
| 1. What was the date of the first thoracic CT-scan for a (suspected) COVID-19 infection? |  |
| 1. What was the date of the first thoracic CT-scan with a CORADS score of 4 or 5? |  |
| 1. Definitive preoperative COVID-19 status | - Positive - Negative |
| If negative proceed to question **31** | |
| If positive | |
| 1. Did COVID-related complications occur preoperatively? | - Yes - No - Unknown |
| If No/Unknown, proceed to question **31** | |
| If Yes | |
| 1. Which COVID-related complications? | - ARDS - Acute kidneyfailure - Arrhythmias - Thrombo-embolic events - Other |
| If YES on thrombo-embolic event | |
| 1. What kind of thrombo-embolic event? | - Pulmonary embolism - Deep venous thrombosis - Intestinal ischemia - Other |
| 1. Did the patient receive therapeutical anticoagulation before occurrence of thrombo-embolic event? | - Yes, because of a comorbidity - Yes, because of COVID-19 - No |
| 1. Did the patient receive therapeutical anticoagulation after the occurrence of a COVID-19 related thrombo-embolic event? | - Yes - No |
|  | |

**Postoperative**

| 1. Did the patient experience symptoms related to COVID-19 postoperatively? | - Yes - No - Unknown |
| --- | --- |
| If No/Unknown, proceed to question **34** |  |
| If YES |  |
| 1. Which symptoms? | - Cough - Fever - Dypnea - Loss of smell and taste - Other |
| 1. How many days after surgery did the COVID-related symptoms start? |  |
|  | |

**Postoperative COVID tests**

| 1. Was the patient tested on COVID-19 postoperatively? | - Yes - No - Unknown |
| --- | --- |
| If No/Unknown, proceed to question **44.** |  |
| If YES |  |
| 1. Which type of tests did the patient undergo postoperatively? | - RT-PCR - Thoracix X-ray or CT - Serology - Other |
| If RT-PCR |  |
| 1. Number of times the RT-PCR test was performed postoperatively |  |
| 1. What was the date of the first postoperative RT-PCR test? |  |
| 1. What was the date of the first postoperative positive RT-PCR test? |  |
| If thoracic X-ray of CT |  |
| 1. Thoracic X-ray: Where consolidations suspected for COVID-19 visible? | - Not performed - Not suspected - Unilateral - Bilateral - Unknown |
| 1. Thoracic CT-scan – What was the CORADS score? | - Not performed - 1 - 2 - 3 - 4 - 5 - Unknown |
| 1. Number of Thoracic CT-scans made for a (suspected) COVID-19 infection? |  |
| 1. What was the date of the first thoracic CT-scan for a (suspected) COVID-19 infection? |  |
| 1. What was the date of the first thoracic CT-scan with a CORADS score of 4 or 5? |  |
| 1. Definitive preoperative COVID-19 status | - Positive - Negative |
|  | |
| I negative, **this is the end of the additional COVID-19 survey** |  |
| If positive: |  |
| 1. Did COVID-related complications occur postoperatively? | - Yes - No - Unknown |
| If No/Unknown ga naar question **50** |  |
| If Yes |  |
| 1. Which COVID-related complications? | - ARDS - Acute kidneyfailure - Arrhythmias - Thrombo-embolic events - Other |
| If YES on thrombo-embolic event | |
| 1. What kind of thrombo-embolic event? | - Pulmonary embolism - Deep venous thrombosis - Intestinal ischemia - Other |
| 1. Did the patient receive therapeutical anticoagulation before occurrence of thrombo-embolic event? | - Yes, because of a comorbidity - Yes, because of COVID-19 - No |
|  | |
| 1. Did the patient receive therapeutical anticoagulation after the occurrence of a COVID-19 related thrombo-embolic event? | - Yes - No |
|  | |

**ntensive Care Unit**

| 1. Was the patient admitted to the ICU because of a (suspected) COVID-19 infection? | - Yes - No - Unknown |
| --- | --- |
|  | |
| If Yes | |
| 1. What kind of respiratory support did the patient receive during ICU-admission? | - None - Low-flow (nasal cannula) - High-flow (non-rebreather mask) - Invasive (intubation) - Unknown |
| 1. Did the patient receive prone-positioned ventilation? | - Yes - No - Unknown |
| 1. Did the patient receive ECMO therapy? | - Yes - No - Unknown |
| 1. ICU admission date |  |
| 1. ICU discharge date |  |
| 1. Total length of ICU admission (days) |  |
|  | |
| 1. Discharge destination after ICU admission | - Nursing ward (same hospital) - Coronary Care Unit - Recovery / medium care unit - Nursing ward (other hospital) - Coronary Care Unit (other hospital) - Recovery / medium care unit (other hospital) - Morgue - Home - Other |
|  | |

**Appendix S5 - Collaborating centers per audit**

|  | **DATO** | **DCRA** | **DHBA** | **DHFA** | **DLCA** | **DSAA** | **DPCA** | **DUCA** |
| --- | --- | --- | --- | --- | --- | --- | --- | --- |
| Albert Schweitzer Ziekenhuis |  |  | x |  | x |  |  |  |
| Antoni van Leeuwenhoek Ziekenhuis - Nederlands Kanker Instituut (AvL-NKI) |  |  | x |  | x |  |  | x |
| Bravis Ziekenhuis |  |  |  |  | x |  |  |  |
| Erasmus Medisch Centrum |  |  | x |  | x |  |  | x |
| Gelre Ziekenhuizen, Apeldoorn |  | x | x |  | x | x |  | x |
| Haaglanden Medisch Centrum |  |  |  |  | x | x |  |  |
| Leids Universitair Medisch Centrum (LUMC) |  |  | x | x | x |  | x | x |
| Maxima Medisch Centrum |  | x | x |  | x | x |  |  |
| Medisch Spectrum Twente |  |  |  | x | x | x |  |  |
| Universitair Medisch Centrum St. Radboud |  |  | x |  | x | x | x | x |
| Amsterdam UMC | x |  |  |  | x | x |  | x |
| Catharina Ziekenhuis | x |  |  |  |  |  | x | x |
| Elisabeth Tweesteden Ziekenhuis | x |  |  |  |  |  |  | x |
| Martini Ziekenhuis |  | x |  |  |  | x |  | x |
| Medisch Centrum Leeuwarden |  |  |  |  |  |  |  | x |
| Universitair Medisch Centrum Groningen (UMCG) |  | x | x | x |  |  | x | x |
| Universitair Medisch Centrum Utrecht |  |  |  |  |  | x |  | x |
| Ziekenhuisgroep Twente |  |  |  | x |  | x |  | x |
| Zuyderland Medisch centrum |  |  |  |  |  |  |  | x |
| Maastricht Universitair Medisch Centrum |  | x | x |  |  | x | x |  |
| Isala Klinieken |  |  | x |  |  | x | x |  |
| Sint Antonius Ziekenhuis, Nieuwegein |  |  |  |  |  | x | x |  |
| Amphia ziekenhuis |  |  | x |  |  |  |  |  |
| St Jansdal Ziekenhuis |  | x | x |  |  |  |  |  |
| Stichting Onze Lieve Vrouwe Gasthuis |  |  | x |  |  |  |  |  |
| Bernhoven |  | x |  | x |  |  |  |  |
| Deventer Ziekenhuis |  | x |  |  |  |  |  |  |
| Groene Hart Ziekenhuis |  | x |  |  |  |  |  |  |
| Jeroen Bosch Ziekenhuis |  | x |  | x |  |  |  |  |
| Maasziekenhuis (Pantein) |  | x |  | x |  |  |  |  |
| Meander Medisch Centrum |  | x |  |  |  | x |  |  |
| Nij Smellinghe |  | x |  |  |  |  |  |  |
| Rode Kruis Ziekenhuis | x | x |  | x |  |  |  |  |
| VieCuri Medisch Centrum |  | x |  |  |  | x |  |  |
| Westfriesgasthuis / Dijklander ziekenhuis |  | x |  |  |  |  |  |  |
| Zaans Medisch Centrum |  | x |  | x |  |  |  |  |
| Admiraal De Ruyter Ziekenhuis |  |  |  |  |  | x |  |  |
| Canisius-Wilhelmina Ziekenhuis |  |  |  |  |  | x |  |  |
| Diakonessenhuis Utrecht |  |  |  |  |  | x |  |  |
| Franciscus Gasthuis & Vlietland |  |  |  |  |  | x |  |  |
| Gelderse Vallei |  |  |  |  |  | x |  |  |
| Haga Ziekenhuis |  |  |  |  |  | x |  |  |
| Maasstad ziekenhuis |  |  |  |  |  | x |  |  |
| Reinier De Graaf Groep |  |  |  |  |  | x |  |  |
| Rijnstate Ziekenhuis |  |  |  |  |  | x |  |  |
| Sint Anna Ziekenhuis |  |  |  |  |  | x |  |  |
| Wilhelmina Ziekenhuis |  |  |  |  |  | x |  |  |
| Slingeland Ziekenhuis |  |  |  |  |  | x |  |  |
| Nederlandse Obesitas Kliniek West, Den Haag | x |  |  |  |  |  |  |  |
| Nederlandse Obesitas Kliniek West, Gouda | x |  |  |  |  |  |  |  |
| Overview of medical centers participating for the specific clinical audits are indicated by x. Abbreviations: DLCA = Dutch Lung Cancer Audit; DUCA = Dutch Upper Gastrointestinal Cancer Audit; DPCA = Dutch Pancreatic Cancer audit; DHBA = Dutch Hepato Biliary Audit; DCRA = Dutch Colorectal Cancer Audit; DHFA = Dutch Hip Fracture Audit; DSAA = Dutch Surgical Aneurysm Audit; DATO = Dutch Audit for Treatment of Obesity | | | | | | | | |
